# A Multi-Strategy Improved Northern Goshawk Optimization Algorithm for Optimizing Engineering Problems

**DOI:** 10.3390/biomimetics9090561

**Published:** 2024-09-16

**Authors:** Haijun Liu, Jian Xiao, Yuan Yao, Shiyi Zhu, Yi Chen, Rui Zhou, Yan Ma, Maofa Wang, Kunpeng Zhang

**Affiliations:** 1 School of Emergency Management, Institute of Disaster Prevention, Langfang 065201, China; liuhaijun@cidp.edu.cn (H.L.); 23661630@st.cidp.edu.cn (J.X.); 22661354@st.cidp.edu.cn (Y.C.); 23661616@st.cidp.edu.cn (R.Z.); 23661605@st.cidp.edu.cn (Y.M.); 2 Institute of Mineral Resources Research, China Metallurgical Geology Bureau, Beijing 101300, China; yaoyuan@cmgb.cn; 3 College of General Education, Hainan Vocational University, Haikou 570216, China; 4 Guangxi Key Laboratory of Trusted Software, Guilin University of Electronic Technology, Guilin 541004, China; wangmaofa2008@126.com; 5 College of Computer Science and Technology, Jilin University, Changchun 130012, China; zhangkp18@mails.jlu.edu.cn

**Keywords:** northern goshawk optimization, cubic mapping strategy, weighted stochastic difference mutation strategy, weighted sine and cosine optimization strategy

## Abstract

Northern Goshawk Optimization (NGO) is an efficient optimization algorithm, but it has the drawbacks of easily falling into local optima and slow convergence. Aiming at these drawbacks, an improved NGO algorithm named the Multi-Strategy Improved Northern Goshawk Optimization (MSINGO) algorithm was proposed by adding the cubic mapping strategy, a novel weighted stochastic difference mutation strategy, and weighted sine and cosine optimization strategy to the original NGO. To verify the performance of MSINGO, a set of comparative experiments were performed with five highly cited and six recently proposed metaheuristic algorithms on the CEC2017 test functions. Comparative experimental results show that in the vast majority of cases, MSINGO’s exploitation ability, exploration ability, local optimal avoidance ability, and scalability are superior to those of competitive algorithms. Finally, six real world engineering problems demonstrated the merits and potential of MSINGO.

## 1. Introduction

In the real-world, there are optimization problems in many fields [1], such as medicine [2,3], transportation [4,5], engineering design [6,7], economics [8], feature selection [9,10,11], artificial neural networks [12,13,14], and so on. The solution of optimization problems can be approached in two principal ways. The first is traditional mathematical methods, and the second is metaheuristic algorithms [15]. Traditional mathematical methods are deterministic methods, including gradient descent [16], Newton’s method [17], conjugate gradient [18], and so on. They solve the problem by using the derivative information of the objective function, which is an effective solution to a continuously differentiable problem [19]. However, when the optimization problem is higher dimensional, non-differentiable, and has multiple local optimal solutions, the traditional mathematical method loses its function [20]. Hence, metaheuristic algorithms have been extensively studied. Metaheuristic algorithms are stochastic methods that consist mainly of two stages: exploration and exploitation phases. In the exploration phase, the algorithm searches for solutions on a global scale to avoid falling into local optima. In the exploitation phase, the algorithm searches in a local area to find a better solution. Metaheuristic algorithms are widely used to solve complex optimization problems in the real world due to their simplicity, flexibility, lack of a deduction mechanism, and ability to avoid local optima.

Metaheuristic algorithms are generally classified into four categories [21]: (1) evolution-based algorithms; (2) swarm-based algorithms; (3) physics- or chemistry-based algorithms; and (4) social- or human-based algorithms. Some well-known and recently metaheuristic algorithms are summarized in Table 1.

Evolution-based metaheuristic algorithms are mainly implemented by modeling the principles of species evolution in nature. An evolution-based metaheuristic algorithm usually consists of three operations: selection, crossover, and mutation. The Genetic Algorithm (GA) [22] and Differential Evolution (DE) [23] are two well-known evolution-based metaheuristic algorithms. The GA was first proposed by Holland in 1975, inspired by Darwin’s theory of natural competition. DE was proposed by Storn and Price in 1997, known for its simplicity, ease of implementation, fast convergence, and robustness. Other evolution-based algorithms include Evolutionary Programming (EP) [24], Genetic Programming (GP) [25], Biogeography-Based Optimization (BBO) [26], Memetic Algorithms (MAs) [27], and Imperialist Competitive Algorithms (ICAs) [28].

Swarm-based metaheuristic algorithms are primarily inspired from the behavior of a group, using the interaction and cooperation of individual sources of information between populations to find the global optimal solution. Particle Swarm Optimization (PSO) is one of the most famous swarm-based algorithms [29] mimicking the flocking behavior of birds. Ant Colony Optimization (ACO) [30] is another popular swarm-based metaheuristic algorithm, inspired from the behavior of ants searching for the shortest path during their foraging process. Other swarm-based metaheuristic algorithms include the Whale Optimization Algorithm (WOA) [31], Cuckoo Search Algorithm (CSA) [32], Grey Wolf Optimizer (GWO) [33], Moth Flame Optimizer (MFO) [21], Sparrow Search Algorithm (SSA) [34], Dung Beetle Optimizer (DBO) [35], Beluga Whale Optimization (BWO) [19], Red Fox Optimization Algorithm (RFO) [36], Sea-horse Optimizer (SHO) [37], Coati Optimization Algorithm (COA) [38], Spider Wasp Optimizer (SWO) [39], Cleaner fish optimization (CFO) [40], and so on.

Physics- or chemistry-based metaheuristic algorithms are based on the simulation of various laws or phenomena in physics or chemistry. One of the well-known algorithms in this category is Simulated Annealing (SA) [41], which is inspired by the physical law of metal cooling and annealing. Other physics- or chemistry-based algorithms include the Gravitational Search Algorithm (GSA) [42], Artificial Chemical Reaction Optimization (ACRO) [43], Sine Cosine Optimization (SCA) [44], Thermal Exchange Optimization (TEO) [45], and the Kepler Optimization Algorithm (KOA) [46].

Social- or human-based algorithms mainly imitate human behaviors. Teaching and Learning Based Optimization (TLBO) [47] is a typical example of this category. It is derived from the behavior of teaching and learning. Other famous or recent social- or human-based algorithms include the Cultural Evolution Algorithm (CEA) [48], Social Learning Optimization Algorithm (SLOA) [49], Socio Evolution and Learning Optimization Algorithm (SELO) [50], and Volleyball Premier League Algorithm (VPL) [51].

**Table 1:** Metaheuristic algorithms.

Category	Algorithms	Authors	Year
Evolutionary	Genetic Algorithm (GA) [22]	Holland	1992
	Genetic Programming (GP) [25]	Koza et al.	1994
	Differential Evolution (DE) [23]	Storn and Price	1997
	Evolutionary Programming (EP) [24]	Yao et al.	1999
	Memetic Algorithms (MAs) [27]	Moscato	2003
	Imperialist Competitive Algorithms (ICAs) [28]	Atashpaz et al.	2007
	Biogeography-Based Optimization (BBO) [26]	Simon	2008
Swarm	Particle Swarm Optimization (PSO) [29]	Kennedy and Eberhart	1995
	Ant Colony Optimization (ACO) [30]	Dorigo et al.	1999
	Cuckoo Search Algorithm (CSA) [32]	Yang and Deb	2009
	Grey Wolf Optimizer (GWO) [33]	Mirjalili et al.	2014
	Moth Flame Optimizer (MFO) [21]	Mirjalili and Seyedali	2015
	Whale Optimization Algorithm (WOA) [31]	Mirjalili et al.	2016
	Seagull Optimization Algorithm (SOA)[52]	Dhiman and Kumar	2019
	Sparrow Search Algorithm (SSA) [34]	Xue et al.	2020
	Red Fox Optimization Algorithm (RFO) [36]	Połap et al.	2021
	Northern Goshawk Optimization (NGO) [53]	Dehghani et al.	2021
	Pelican Optimization Algorithm (POA) [54]	Trojovský and Dehghani	2022
	Golden Jackal Optimization (GJO) [55]	Chopra and Ansari	2022
	Beluga Whale Optimization (BWO) [19]	Zhong et al.	2022
	Sea-horse Optimizer (SHO) [37]	Zhao et al.	2023
	Dung Beetle Optimizer (DBO) [35]	Xue et al.	2023
	Coati Optimization Algorithm (COA) [38]	Dehghani et al.	2023
	Spider Wasp Optimizer (SWO) [39]	Basset et al.	2023
	Cleaner fish optimization (CFO) [40]	Zhang et al.	2024
Physics and Chemistry	Simulated Annealing (SA) [41]	Kirkpatrick et al.	1983
	Magnetic Optimization Algorithm (MOA) [56]	Tayaraniet al.	2008
	Gravitational Search Algorithm (GSA) [42]	Rashedi et al.	2009
	Artificial Chemical Reaction Optimization (ACRO) [43]	Alatas	2011
	Lightning Search Algorithm (LSA) [57]	Mirjalili	2015
	Sine Cosine Optimization (SCA) [44]	Tanyildizi et al.	2016
	Golden Sine Algorithm (GSA) [58]	Kaveh et al.	2017
	Thermal Exchange Optimization (TEO) [45]	Abualigah et al.	2017
	Kepler Optimization Algorithm (KOA) [46]	Basset et al.	2023
Human	Teaching and Learning Based Optimization (TLBO) [47]	Rao et al.	2011
	Cultural Evolution Algorithm (CEA) [48]	Kuo and Lin	2013
	Election Algorithm (EA) [59]	Emami et al.	2015
	Social Learning Optimization Algorithm (SLOA) [49]	Liu et al.	2017
	Socio Evolution and Learning Optimization Algorithm (SELO) [50]	Kumar et al.	2018
	Volleyball Premier League Algorithm (VPL) [51]	Moghdani et al.	2018

As mentioned earlier, a large number of metaheuristic algorithms have been developed. However, the No Free Lunch (NFL) theorem states that no metaheuristic optimization algorithm can solve all optimization problems [60]. As real-world problems become increasingly challenging, it is necessary to improve existing optimization algorithms to design more effective and efficient optimization algorithms for increasingly complex optimization problems in the real world. Northern Goshawk Optimization (NGO) [53], inspired by the predatory behavior of the northern goshawk, has gained a lot of attention shortly after its first proposal. For example, Chang et al. [61] used NGO to optimize the life-cycle costs of the power grid. El-Dabah et al. [62] utilized NGO to optimize the parameters in PV modules. Wu et al. [63] proposed a deep learning model CNN-LSTM for predicting PV power, where NGO was used to optimize CNN-LSTM. Although the NGO algorithm has achieved some accomplishments, it still has some drawbacks: (1) the initial population is randomly generated and lacks diversity; (2) it exhibits slow convergence speed; (3) it easily falls into local optima.

To address the issues in the original NGO algorithm, this paper adds three strategies to it and proposes a Multi-Strategy Improved Northern Goshawk Optimization, called MSINGO. Then, the improved MSINGO is used to optimize six engineering problems.

The main contributions of this paper are summarized as follows:

(1) To enhance the diversity of the initial population, this paper adds the cubic mapping strategy in the initialization phase of the original NGO algorithm;
(2) To avoid NGO being trapped in local optima, a novel weighted stochastic difference variation strategy is introduced in the exploration phase. It will help NGO jump out of local optima;
(3) To accelerate the convergence speed, a weighted sine and cosine optimization strategy is added in the exploitation phase of the original NGO algorithm;
(4) To evaluate the performance of our improved MSINGO, we compare it with five highly cited and six recently proposed metaheuristic algorithms on CEC2017 test functions and six engineering design problems.

The rest of this paper is structured as follows. Section 2 briefly introduces the original NGO. Section 3 describes the improved MSINGO in detail. Section 4 shows experiments on 29 benchmark functions on CEC2017. Section 5 evaluates the performance of improved MSINGO in solving six engineering problems. Section 6 summarizes this paper.

## 2. Overview of Original NGO

The mathematical model of the NGO algorithm is briefly presented in this section. NGO mainly simulates the predatory process of the northern goshawk. The process of NGO involves 3 main phases: initialization, the exploration phase and the exploitation phase, which will be described in detail below.

### 2.1. Initialization

NGO is a population-based algorithm. Each individual northern goshawk in the population is considered as a candidate solution. In the initial stage of the NGO algorithm, candidate solutions are randomly generated, as shown in Equation (1). All candidate solutions form the population matrix, as shown in Equation (2).

(1) $$x_{i,j}=lb_{j}+r\times \left(ub_{j}-lb_{j}\right),i=1,2,\cdots ,N,j=1,2,\cdots ,dim$$

(2) $$\;X={\left[\begin{array}{c} X_{1}\\ \vdots \\ X_{i}\\ \vdots \\ X_{N} \end{array}\right]}_{N\times m}={\left[\begin{array}{ccccc} x_{1,1} & \cdots & x_{1,d} & \cdots & x_{1,dim}\\ \vdots & \ddots & \vdots & \vdots & \vdots \\ x_{i,1} & \cdots & x_{i,d} & \cdots & x_{i,dim}\\ \vdots & \vdots & \vdots & \ddots & \vdots \\ x_{N,1} & \cdots & x_{N,d} & \cdots & x_{N,dim} \end{array}\right]}_{N\times dim}$$

where X represents the population matrix, storing positions of all possible candidate solutions; $X_{i}$ is the position of the ith candidate solution, which will be updated during optimization; $x_{i,j}$ indicates the jth dimension of the ith solution; r is a random real number in the interval [0, 1]; ${lb}_{j}$ and ${ub}_{j}$ are the lower bound and upper bound of the jth variable, respectively; N denotes the size of the population; dim represents the dimension to be optimized.

In the optimization process, the objective function is used to calculate the fitness value of each candidate solution. All fitness values are stored in the fitness matrix F(X), as shown in Equation (3). The candidate solution with the minimum fitness is the optimal solution.

(3) $$\;{\left.F\left(X\right)=\left[\begin{array}{c} F_{1}=F\left(X_{1}\right)\\ \vdots \\ F_{i}=F\left(X_{i}\right)\\ \vdots \\ F_{N}=F\left(X_{N}\right) \end{array}\right.\right]}_{N\times 1}$$

where F is the objective function, and $F_{i}$ is the fitness value obtained via the objective function with the ith proposed solution.

### 2.2. Exploration Phase

The exploration phase of NGO imitated a northern goshawk randomly selecting prey in the search space and attacking it. In this phase, the mathematical model is shown in Equations (4) and (5)

(4) $$X_{i}^{t+1}=\left\{\begin{array}{c} X_{i}^{t}+r\times \left(X_{k}^{t}-I\times X_{i}^{t}\right),{\;\;\;\;\;\;\;\;F}_{k}^{t}<F_{i}^{t}\\ X_{i}^{t}+r\times \left(X_{i}^{t}-X_{k}^{t}\right),{\;\;\;\;\;\;\;\;F}_{k}^{t}\geq F_{i}^{t} \end{array}\right.$$

(5) $$X_{i}^{t}=\left\{\begin{array}{c} X_{i}^{t+1},\;\;\;\;F_{i}^{t+1}<F_{i}^{t}\\ X_{i}^{t},\;\;\;\;F_{i}^{t+1}\geq F_{i}^{t} \end{array}\right.$$

where k represents a random natural number in the interval [1,N]; $X_{k}^{t}$ is the position of the *k*th solution in the tth iteration; $X_{i}^{t}$ is the position of the ith solution in the tth iteration; $X_{i}^{t+1}$ is the position of the *i*th solution in iteration t+1; $F_{k}^{t}$ is the fitness value of the kth solution of the objective function in the *t*th iteration; $F_{i}^{t}$ is the fitness value of the ith solution of the objective function in the *t*th iteration; $F_{i}^{t+1}$ is the fitness value of the *i*th solution of the objective function in iteration t+1; r is a random number in the interval [0, 1]; I is a random number of 1 or 2.

### 2.3. Exploitation Phase

The exploitation phase of the NGO algorithm mimicked the behavior of a northern goshawk chasing and hunting prey. In this phase, the positions are updated using Equations (6)–(8).

(6) $$\;X_{i}^{t+1}=X_{i}^{t}+R\times \left(2\times r-1\right)\times X_{i}^{t}$$

(7) $$R=0.02\times \left(1-\frac{t}{T}\right)$$

(8) $$X_{i}^{t}=\left\{\begin{array}{c} X_{i}^{t+1},\;\;\;\;F_{i}^{t+1}<F_{i}^{t}\\ X_{i}^{t},\;\;\;\;F_{i}^{t+1}\geq F_{i}^{t} \end{array}\right.$$

where t is the current number of iteration, and T is the maximum number of iterations; r is a random number in the interval [0, 1].

## 3. Our Proposed MSINGO

We proposed a Multi-Strategy Improved NGO(MSINGO) by adding three strategies to the original NGO algorithm. In this section, three strategies (i.e., cubic mapping, weighted random difference variation, and weighted sine and cosine optimization) and the proposed MSINGO will be introduced below.

### 3.1. Cubic Mapping Strategy

In the original NGO algorithm, the initial population is randomly generated, which may lead to insufficient population diversity. Many researchers have proved that chaotic mapping can improve population diversity, making optimization algorithm find the global optimal solution easier [64,65]. Cubic mapping is a common form of chaotic mapping [66]. We use it to initialize the population, which will enhance the population’s diversity. The mathematical model of cubic mapping is shown in Equation (9).

(9) $$z_{p+1}=a\times z_{p}\times \left(1-{z_{p}}^{2}\right),\;p=0,\;1,\;2,\cdots ,N\;*\;dim$$

where a is a parameter set to 2.595; $z_{p}$ is the pth chaotic value, with $z_{0}$ = 0.3; N denotes the population size; dim indicates the dimension of problem variables to be optimized.

The new population initialization formula with the cubic mapping strategy is shown in Equation (10).

(10) $$x_{i,j}=lb_{j}+z_{p+1}\times \left(ub_{j}-lb_{j}\right),i=1,\;2,\cdots ,N,\;j=1,\;2,\cdots ,\;dim$$

### 3.2. Weighted Stochastic Difference Mutation Strategy

In the exploration phase of the original NGO algorithm, individuals in the population are updated by generating new individuals nearby randomly. In this way, new individuals may be close to the old ones, which can lead to NGO falling into local optima. A mutation strategy can help escape from the local optima. In this paper, we proposed a novel mutation strategy named weighted stochastic differential mutation strategy, which will help NGO jump out of local optima. Our weighted stochastic difference mutation strategy consists of two parts: differential variation value Q and its weight W.

The differential variation value Q is calculated with Equation (11).

(11) $$\;Q=R_{1}\times \left[X_{best}^{t}-X_{i}^{t}\right]-R_{2}\times \left[X_{random}^{t}-X_{i}^{t}\right]$$

where $X_{best}^{t}$ represents the current optimal position; $X_{random}^{t}$ denotes a randomly selected position; $R_{1}$ and $R_{2}$ are random numbers in the interval [0, 1].

The weight W is generated using the weighted Levy flight technique [67], as shown in Equations (12)–(15).

(12) W=ω×Levy

(13) $$\omega =e^{-{\left(\frac{10t}{T}\right)}^{2}}+10$$

(14) $$Levy=s\times \frac{u\times \sigma }{{\left|v\right|}^{\frac{1}{\eta }}}$$

(15) $$\;\sigma ={\left(\frac{\Gamma \left(1+\eta \right)\times \sin\left(\frac{\pi \eta }{2}\right)}{\Gamma \left(\frac{1+\eta }{2}\right)\times \eta \times 2\left(\frac{\eta -1}{2}\right)}\right)}^{\frac{1}{\eta }}$$

Among them, t is the current number of iterations, and T is the maximum number of iterations; s is 0.05; η is 1.5; u and v are normally distributed random numbers; Γ is the gamma function.

Then, the weighted differential variation value is calculated with Equation (16).

(16) WR=W×Q

Finally, we replaced the original Equation (4) in the NGO with the new Equation (17).

(17) $${\;X}_{i}^{t+1}=\left\{\begin{array}{c} X_{i}^{t}+r\times \left(X_{k}^{t}-I{\times X}_{i}^{t}\right)+WR,{\;\;\;\;\;\;\;\;F}_{k}^{t}<F_{i}^{t}\\ X_{i}^{t}+r\times \left(X_{i}^{t}-X_{k}^{t}\right)+WR,{\;\;\;\;\;\;\;\;\;\;\;\;\;\;\;F}_{k}^{t}\geq F_{i}^{t} \end{array}\right.$$

### 3.3. Weighted Sine and Cosine Optimization Strategy

The convergence speed of the original algorithm is slow. To solve this problem, we add the weighted sine and cosine optimization strategy to the exploitation phase. The mathematical model of the sine and cosine optimization strategy is shown in Equation (18).

(18) $$X_{i}^{t+1}=\left\{\begin{array}{c} X_{i}^{t}+r_{1}\times \sin\left(r_{2}\right)\times \left|r_{3}\times X_{best}^{t}-X_{i}^{t}\right|,{\;\;\;\;\;r}_{4}<0.5\\ X_{i}^{t}+r_{1}\times \cos\left(r_{2}\right)\times \left|r_{3}\times X_{best}^{t}-X_{i}^{t}\right|,{\;\;\;\;\;r}_{4}\geq 0.5 \end{array}\right.$$

where r1=2 ∗ ((1−t/T)^2); $r_{2}$ represents a random number in the interval [0, 2π]; $r_{3}$ is a random number in the interval [0, 2], $r_{4}=r\;*\;(1-0.5\;*\;t/T)$; r is a random number between 0 and 1.

Finally, we use Equation (19) instead of Equation (6) as the position updating formula in the exploitation phase.

(19) $$X_{i}^{t+1}=\left\{\begin{array}{c} X_{i}^{t}+r_{1}\times sin{(r}_{2})\times \left|r_{3}\times X_{best}^{t}-X_{i}^{t}\right|+W,{\;\;\;\;\;r}_{4}<0.5\\ X_{i}^{t}+r_{1}\times cos{(r}_{2})\times \left|r_{3}\times X_{best}^{t}-X_{i}^{t}\right|+W,{\;\;\;\;\;r}_{4}\geq 0.5 \end{array}\right.$$

### 3.4. The Detail of Our Proposed MSINGO

**Figure 1:**
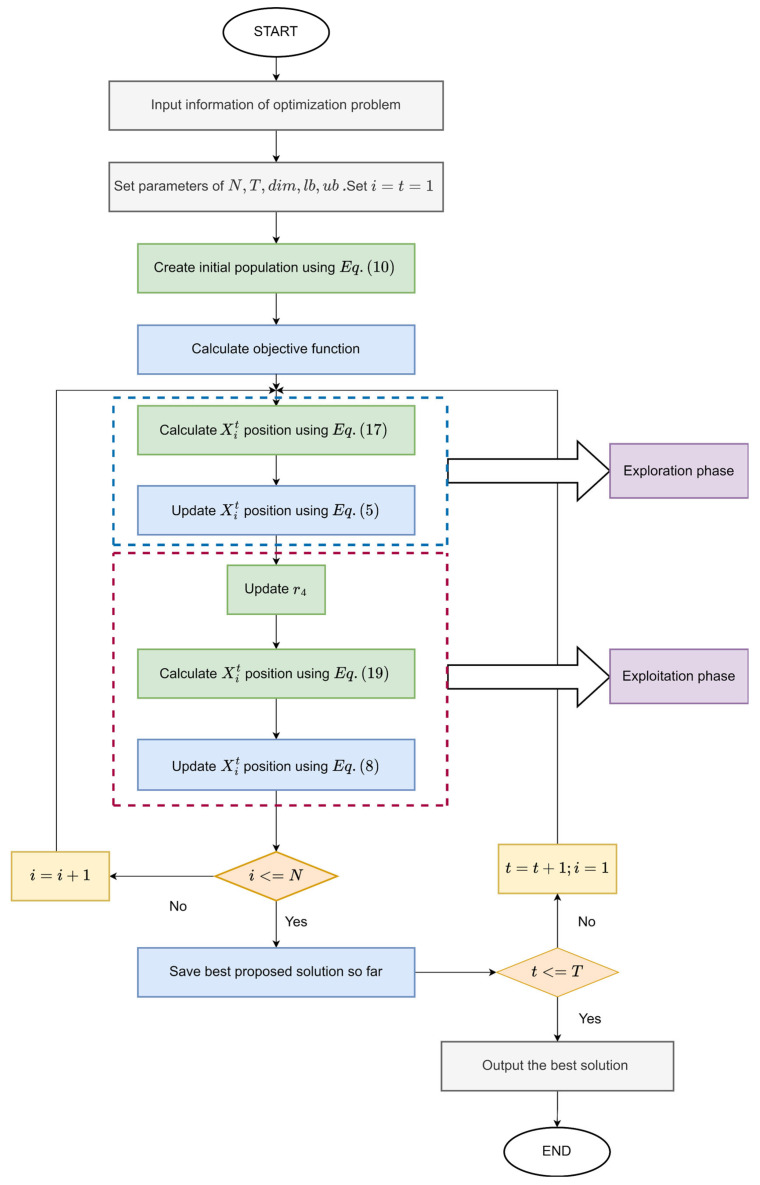
The flow chart of the MSINGO algorithm.

The pseudo-code of the proposed MSINGO algorithm is shown in Algorithm 1. The flow chart of MSINGO is shown in Figure 1, where the green parts are our improvements.

The pseudo-code of the MSINGO algorithm.
**Algorithm 1.** Pseudo-code of the MSINGO algorithm**Input:**The initial parameters of MSINGO, including the maximum number of iterations T, the number of population members N, the dimension of problem variables dim, the lower bound and upper bound of problem variables lb, ub.**Output:**Optimal fitness value1:Set i=1, t=1
2:Create initial population using **Equation (10)**3:**While** t ≤ T **Do**4:  **While** i ≤ N **Do**5:  Exploration phase:6:    Calculate the position of the ith solution using **Equation (17)**7:    Update the position of the ith solution using **Equation (5)**8:  Exploitation phase:9:    Calculate $r_{4}$
10:    Calculate the position of the ith solution using **Equation (19)**11:    Update the position of the ith solution using **Equation (8)**12:  
**End While**
13:  Save best proposed solution so far14:  
t=t+1
15:**End While**16:Output the best solution

### 3.5. Time Complexity Analysis

The time complexity of the MSINGO algorithm consists of three parts: population initialization, exploration phase, and exploitation phase. The primary parameters that affect time complexity are the maximum number of iterations *T*, the dimension to be optimized dim, and the size of population *N*. First of all, in the population initialization, the time complexity of MSINGO is O(N ∗ dim). Secondly, in the iteration process, each individual will be updated in the exploration and exploitation phases. Therefore, the time complexity in the two phases is O(T ∗ N ∗ dim), respectively. Consequently, the total time complexity of MSINGO is O((2 ∗ T+1) ∗ dim ∗ N).

## 4. Experimental Results and Analysis

In this section, we evaluated the performance of the proposed MSINGO on the CEC2017 test functions. The whole experiment consists of the following four parts: (1) the impact of three strategies on NGOs; (2) qualitative analysis of the MSINGO algorithm; (3) comparison with 11 well-known metaheuristic algorithms; and (4) scalability analysis of the MSINGO algorithm. All experiments were tested in a computer with Intel (R) Core (TM) i7-8565U,1.80 GHz CPU (Intel Corporation. City, Santa Clara, CA, USA). And the algorithms are all based on Python 3.7.

### 4.1. Benchmark Functions

To evaluate the performance of the MSINGO algorithm, we conducted experiments on 29 benchmark functions of IEEE CEC2017. The CEC2017 test functions contains 2 unimodal functions (C1–C2) to test the exploitation capability, 7 multimodal functions (C3–C9) to test the exploration capability, and 10 hybrid functions (C10–C19) and 10 composition functions (C20–C29) to test the algorithm’s ability to avoid local optima. The detailed descriptions of these benchmark functions are shown in Table 2, where range denotes the boundary of design variables, and $f_{min}$ represents the optimal value.

### 4.2. Competitor Algorithms and Parameters Setting

MSINGO is compared with 5 highly cited algorithms, including DE, MFO, WOA, SCA, and SOA, and 6 recently proposed metaheuristic algorithms, including SSA, DBO, POA, BWO, GJO, and NGO. The parameter settings of all algorithms are shown in Table 3. The population size and the maximum number of iterations of all algorithms are set to 30 and 500, respectively. Furthermore, in order to decrease the impact of the random factor, each algorithm was executed independently 30 times on each test function.

### 4.3. Influence of the Three Mechanisms

This section combines the cubic mapping strategy (C), weighted stochastic difference mutation strategy (WS), and weighted sine and cosine optimization strategy (WSC) with NGO, and analyzes their influence on improving NGO’s performance. The details of these different NGO algorithms are shown in Table 4, where ‘1’ indicates that the strategy is integrated with NGO and ‘0’ indicates the opposite.

Seven kinds of strategic NGO algorithms and the original NGO are tested on CEC2017 test functions, and the dimension to be optimized (dim) is set to 30. We calculated the average (Ave), standard deviation (Std), and highlighted the optimal algorithm in bold. The results are presented in Table 5.

According to the average fitness (Ave) of each algorithm in Table 5, the Friedman test method was carried out to rank the fitness of all the algorithms. The ranking of each algorithm is shown in Table 6, where average rank represents the average ranking of each algorithm and overall rank represents the final ranking of each algorithm, and +/−/= represents the number of test functions for which MSINGO performs better, lower or equal to that of other comparison algorithms. The smaller the values of average rank and overall rank, the better the performance of the algorithm.

According to average rank in Table 6, the eight NGO algorithms are ranked as follows, MSINGO > WS_NGO > C_WS_NGO > WS_WSC_NGO > C_WSC_NGO > WSC_NGO > NGO > C_NGO. It can be seen that MSINGO performs better than WS_NGO on only 15 functions, indicating that the weighted stochastic difference variation (WS) strategy has played a crucial role in improving NGO algorithms. However, according to the average ranking of the algorithm, MSINGO is superior to WS_NGO, indicating that the other two strategies also play an auxiliary role in improving NGO algorithms. Therefore, the performance of the algorithm is improved the most when the three strategies are added simultaneously.

### 4.4. Qualitative Analysis

**Figure 2:**
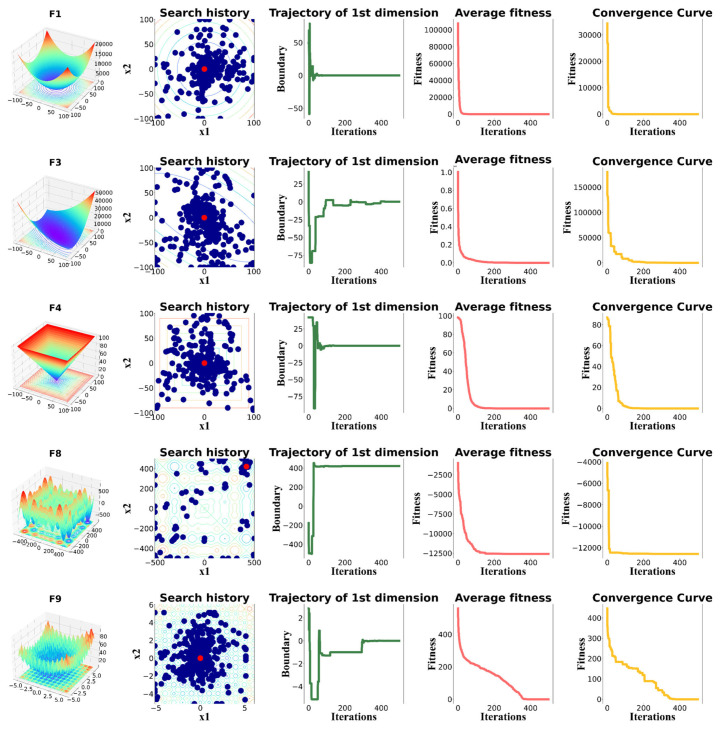

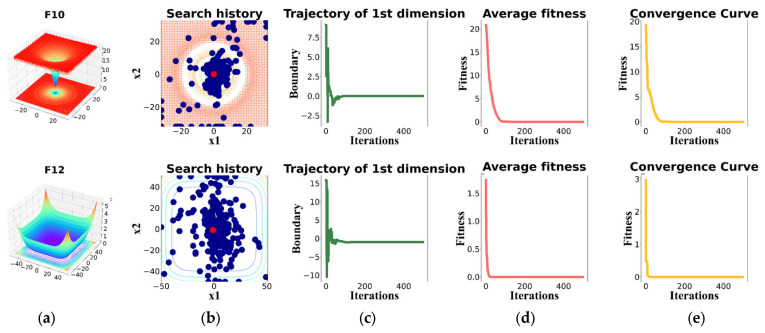
Qualitative results of MSINGO, including (**a**) function’s landscape; (**b**) search history; (**c**) trajectory of 1st dimension; (**d**) average fitness; and (**e**) convergence curve.

The qualitative analysis of MSINGO in solving common unimodal and multimodal functions are shown in Figure 2, and the detail and complete information of these functions are given in [24]. In the qualitative analysis of MSINGO, four well-known indicators are used to intuitively analyze the performance of the MSINGO algorithm, including the following: (1) search history; (2) the trajectory of the first northern goshawk in the 1st dimension; (3) the average fitness of the northern goshawk population; and (4) the convergence curve of the best candidate solution.

The search history shows the position of each northern goshawk in the search space during the iterations. As can be seen in Figure 2b, the red dot in the search history represents the location of the global optimum and the blue dots represent the locations of the candidate best solutions during the iterations. It can be easily seen from the search history that MSINGO can search globally and converge quickly once finding the main optimal area, indicating MSINGO has a good ability to perform global exploration and local exploitation.

The trajectory of the first dimension refers to the position changes of the first northern goshawk in the first dimension, which indicates the primary exploratory behavior of MSINGO. As shown in Figure 2c, in the trajectory diagram of the first northern goshawk, the position of the first northern goshawk experienced rapid oscillations in the initial iterations, indicating that the MSINGO algorithm can quickly identify the main optimal region. In the subsequent iterations, there were slight oscillations, indicating that MSINGO searched around the optimal position and converged to it.

The average fitness curves and convergence curves for different benchmark functions are also provided in Figure 2d,e. Among them, rapid decline occurred in the initial iterations, representing that MSINGO converged quickly.

### 4.5. Comparison with 11 Well-Known Metaheuristic Algorithms

In qualitative analysis, we intuitively demonstrated the exploitation and exploration capabilities of the proposed MSINGO algorithm. In this section, we quantitatively compared our proposed MSINGO algorithm with 11 metaheuristic algorithms (5 highly cited and 6 recently proposed) on CEC2017 test functions. The comparison algorithms are described in Section 4.2.

Comparative experiments include the following: (1) comparison of exploitation capabilities on unimodal benchmark functions (C1–C2); (2) comparison of exploration abilities on multimodal benchmark functions (C3–C9); (3) comparison of local optimal avoidance abilities on hybrid functions (C10–C19) and composition functions (C20–C29).

Furthermore, the Friedman test was used to evaluate overall performance of 12 metaheuristics algorithms. The Wilcoxon signed-rank test was used to verify if two sets of solutions are dissimilar statistically substantial or not.

In these comparative experiments, the dimension to be optimized (dim) was set to 30. For a fair comparison, each algorithm was implemented 30 independent runs in each benchmark and the Ave, Std, and Rank were calculated, where Ave and Std indicate the mean and standard deviation of the optimal values, and Rank presents the algorithm’s ranking. A low Ave indicates a higher optimization performance and a lower Std indicates a more stable optimization performance. The algorithm with the best performance is bolded to highlight.

Below is a detailed discussion of these comparative experiments.

#### 4.5.1. Exploitation Ability Analysis

To test the exploitation ability of MSINGO, we compare it with other 11 metaheuristic algorithms on two unimodal functions (C1–C2). The quantitative results on these two unimodal functions are shown in Table 7. It can be seen from Table 7 that MSINGO ranks first on the two unimodal functions, indicating MSINGO has the best exploitation ability among all the 12 algorithms.

**Figure 3:**
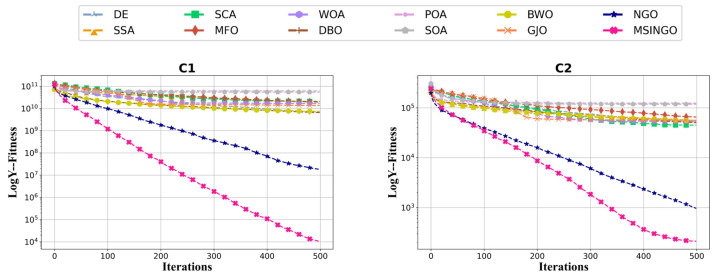
Convergence curves of different algorithms on the unimodal functions (C1–C2, dimension = 30).

The convergence curves of 12 metaheuristic algorithms on C1 and C2 are shown in Figure 3. Obviously, our MSINGO algorithm has the fastest convergence speed and minimum fitness value, which shows that the proposed MSINGO is the most competitive algorithm in these unimodal functions.

**Figure 4:**
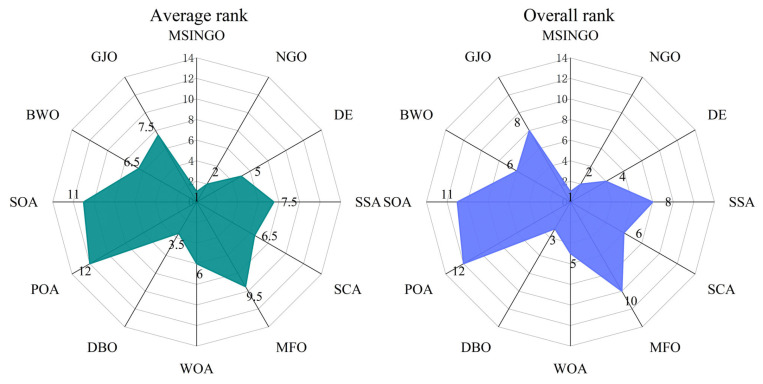
Radar maps of Friedman ranking of different algorithms on unimodal functions.

According to the Rank score in Table 7, the Friedman test was performed, and the result is shown in Figure 4, where average rank represents the average ranking of all algorithms, and overall rank represents the final ranking of all algorithms in unimodal functions. The smaller the average rank and overall rank, the better the performance of the algorithm. As can be seen from Figure 4, MSINGO ranks first, indicating that it is superior to other comparison algorithms in exploitation ability.

The *p*-values obtained via the Wilcoxon signed-rank test between MSINGO and each of the comparison algorithms are presented in Table 8. A *p*-value less than 0.05 indicates a significant difference between the comparison algorithm and MSINGO. Conversely, there is no significant difference. As seen in Table 8, on the two unimodal functions, all *p*-values are less than 0.05, indicating that MSINGO is significantly better than other comparison algorithms.

The results in this section showed that the MSINGO algorithm has the best exploitation ability among all 12 algorithms.

#### 4.5.2. Exploration Ability Analysis

In this section, we tested the exploration ability of MSINGO on seven multimodal functions (C3–C9). Quantitative analysis is presented in Table 9, where MSINGO ranks first on five functions (C3, C4, C5, C8, and C9) and second on two functions (C6 and C7), indicating that MSINGO’s exploration ability is superior to comparative algorithms in the vast majority of cases.

**Figure 5:**
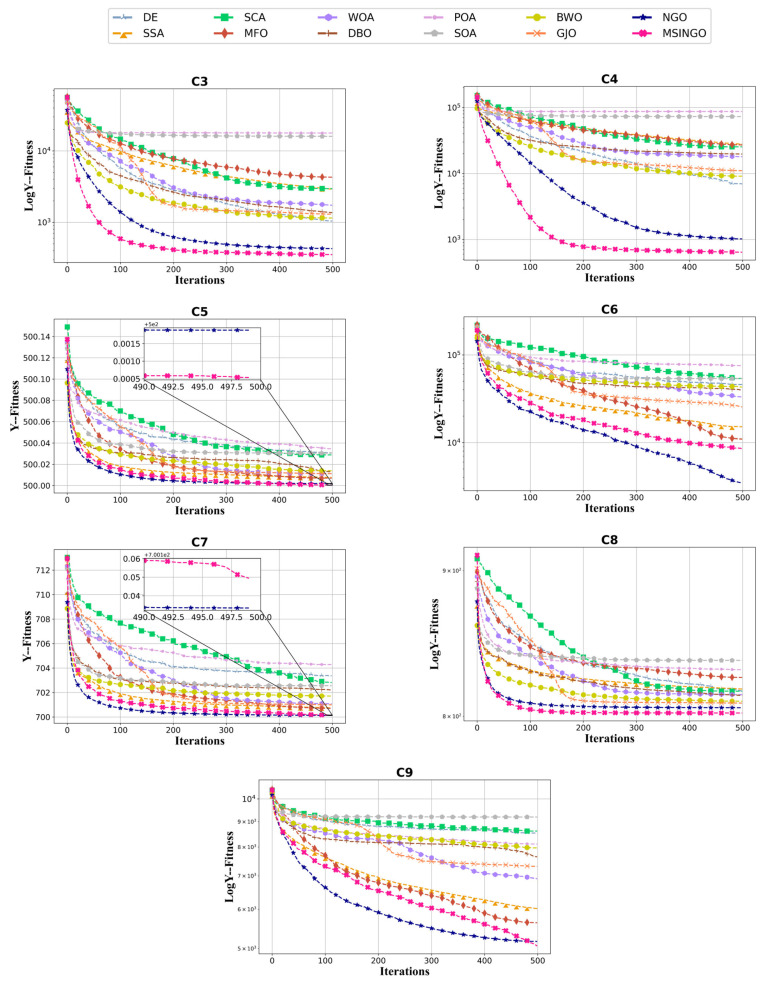
Convergence curves of different algorithms on the multimodal functions (C3–C9, dimension = 30).

The convergence curves from C3 to C9 are shown in Figure 5. It can be seen that in terms of convergence speed, MSINGO ranks first on three functions (C3, C4, and C8) and second on the other four functions; from the perspective of the optimal solution, MSINGO is the smallest among five functions (C3, C4, C5, C8, and C9) and the second smallest among two functions (C6 and C7).

**Figure 6:**
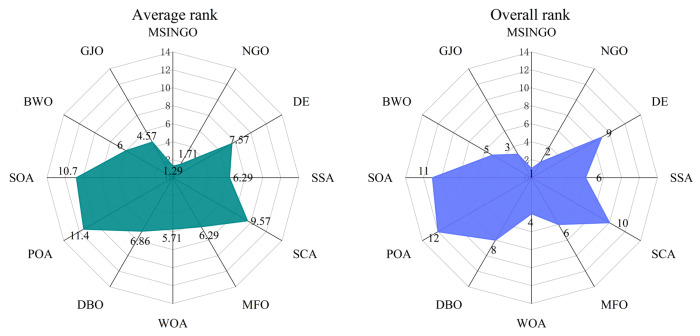
Radar maps of Friedman ranking of different algorithms on multimodal functions.

Friedman test results for these seven multimodal functions are shown in Figure 6, where MSINGO ranks first, indicating it has the best exploration ability among the 12 algorithms.

Furthermore, Table 10 shows the *p*-value results of the Wilcoxon signed-rank test on MSINGO against 11 other algorithms. The vast majority of *p*-values are less than 0.05, indicating that MSINGO is better than the comparison algorithms in terms of exploration capability.

The results of this section indicate that MSINGO is superior to other comparison algorithms in exploration ability.

#### 4.5.3. Local Optimal Avoidance Ability Analysis

In this section, 10 hybrid functions (C10–C19) and 10 composition functions (C20–C29) are selected to verify the ability to avoid local optimal on 12 metaheuristic algorithms. Quantitative statistical results are presented in Table 11 and Table 12. It can be seen that MSINGO ranks first on nineteen functions (C10 to C17 and C19 to C29) and second on C18. These results indicate that MSINGO has superior performance in avoiding local optima compared to 11 other metaheuristic algorithms.

**Figure 7:**
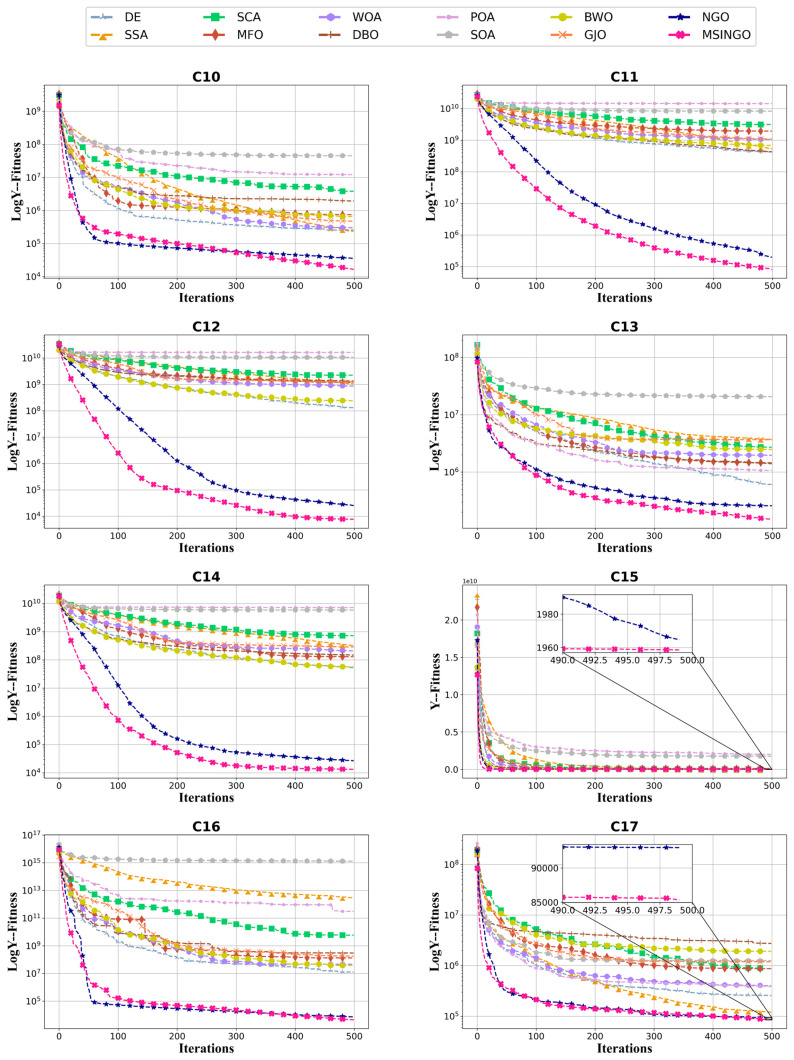

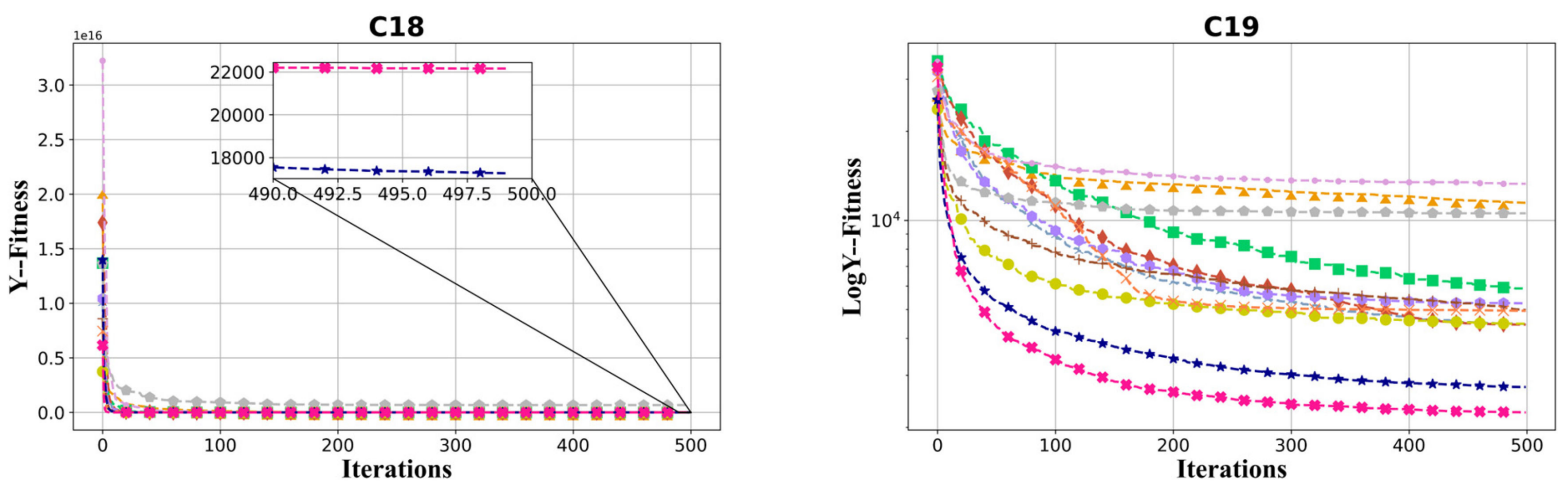
Convergence curves of different algorithms on hybrid functions (C10–C19, dimension = 30).

**Figure 8:**
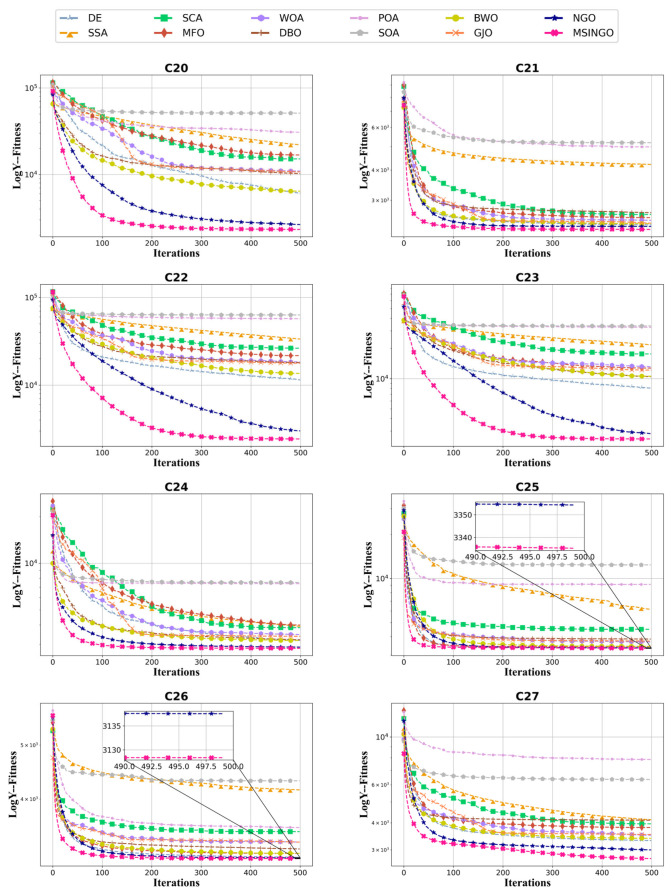

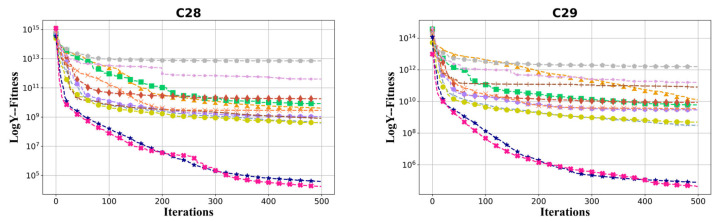
Convergence curves of different algorithms on composition functions (C20–C29, dimension = 30).

The convergence curves of hybrid functions and composition functions are shown in Figure 7 and Figure 8, respectively. For these 20 hybrid and composition functions, MSINGO ranks first on 19 functions (C10–C17 and C19–C29) and second on C18 in convergence speed and optimal solution.

**Figure 9:**
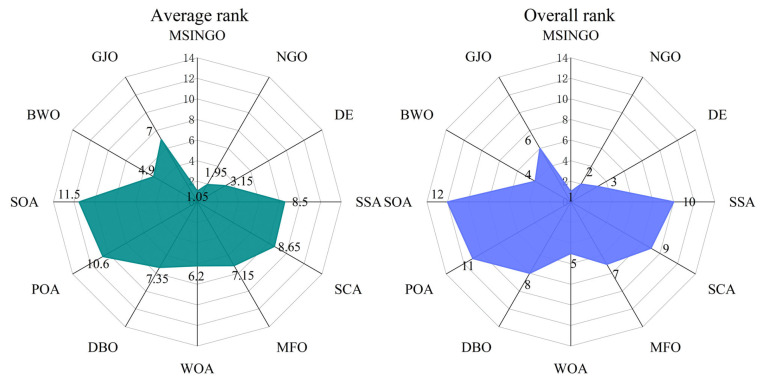
Radar maps of Friedman ranking of different algorithms on hybrid functions and composition functions.

The results of the Friedman test for 20 hybrid and composition functions are shown in Figure 9. It can be seen that MSINGO ranks first among 12 algorithms.

The *p*-values of Wilcoxon signed-rank tests on hybrid functions (C10–C19) and composition functions (C20–C29) are shown in Table 13. Obviously, the majority of the *p*-values are less than 0.05, indicating a significant difference between MSINGO and the majority of algorithms in terms of avoiding local optima.

The results of this section show that MSINGO is the best in avoiding local optima among all algorithms.

**Figure 10:**
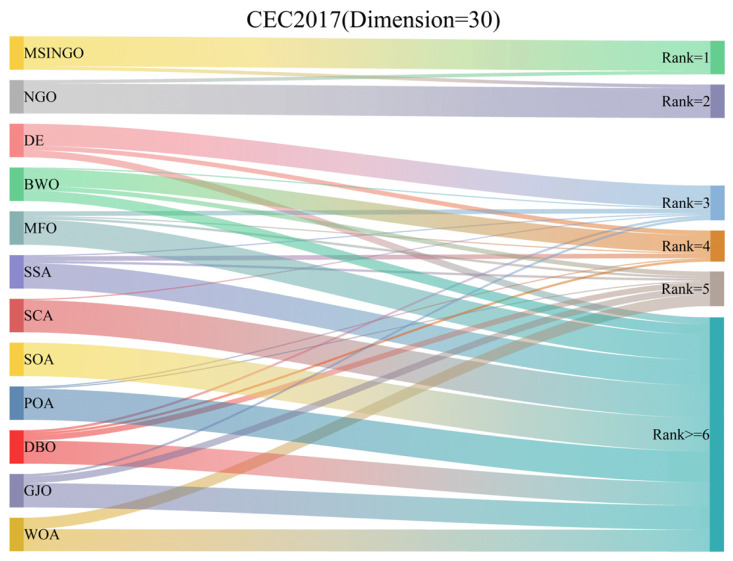
The ranking Sankey of different algorithms on CEC2017 (Dimension = 30).

In Figure 10, we showed the Sankey ranking of the 12 algorithms, demonstrating that our proposed algorithm mostly maintains the first position in different test functions.

Among all the functions, MSINGO and NGO have similar optimal values in the seven functions of C5, C8, C9, C15, C6, C24, and C26, indicating that MSINGO’s advantages are not very obvious among these seven functions. Meanwhile, the optimal values for functions C6, C7, and C18 are not as good as NGO. This shows that the strategy we designed is not optimal, and some adjustments may be needed, such as parameter tuning. But we have obvious advantages in the other functions, so we need to consider the strategy from multiple aspects.

### 4.6. Scalability Analysis

In this section, to test the scalability of MSINGO, the dimension of benchmark function to be optimized (dim) is set to 100. For all algorithms, the population size is set to 30 and the maximum number of iterations is set to 500. Meanwhile, to reduce the influence of random factors, each algorithm was executed independently 30 times on each test function, and the Ave, Std, and Rank were calculated. The algorithm with the best performance is bolded. The results are shown in Table 14.

Among all the algorithms, MSINGO ranked first on 26 test functions (C1 to C5, C8, and C10 to C29), second on C7, fourth on C9, and fifth on C6.

**Figure 11:**
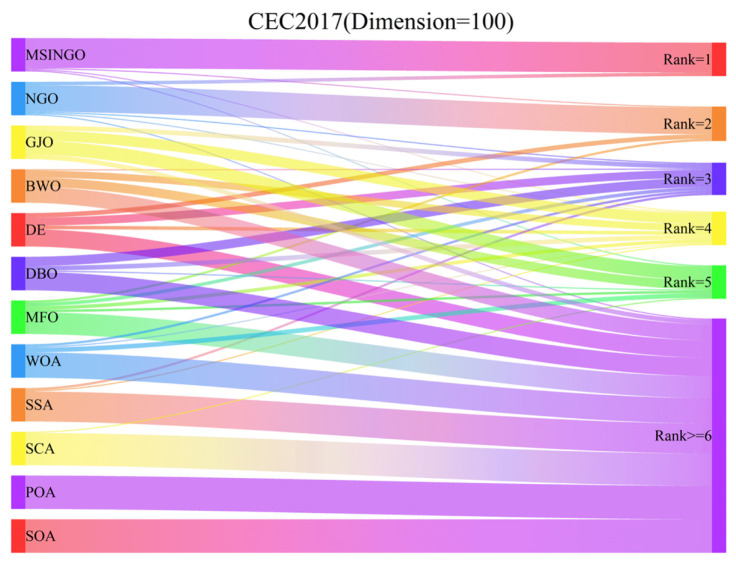
The Sankey ranking of different algorithms on CEC2017 (dimension = 100).

In Figure 11, we showed the Sankey ranking of the 12 algorithms on high-dimensional test functions, demonstrating that our proposed algorithm mostly maintains the first positions in different test functions.

**Figure 12:**
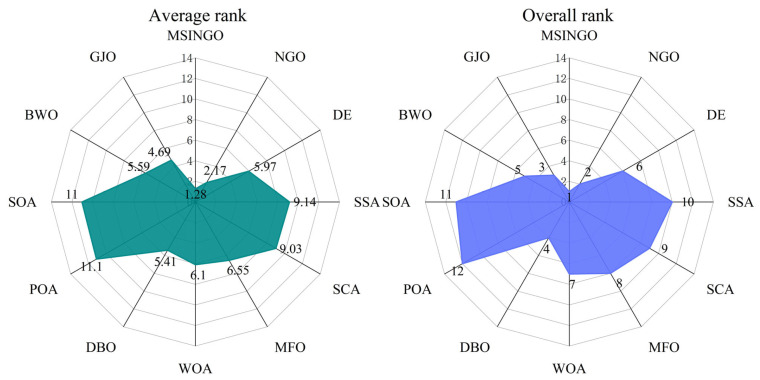
Radar maps of Friedman ranking of different algorithms on all functions.

The results of the Friedman test on all 100-dimensional benchmark functions (C1–C29) are shown in Figure 12. It can be seen that MSINGO ranks first in all algorithms, indicating that MSINGO is the best of the 12 algorithms when dealing with high-dimensional problems.

### 4.7. Memory Occupation

We choose one function from unimodal (C1), multimodal (C4), hybrid function (C15), and composition function (C27) to experiment. The population size and the maximum number of iterations of all algorithms are set to 30 and 500, respectively. And the dimension to be optimized (dim) was set to 30. The results are shown in Table 15. It can be concluded that the memory occupation of MSINGO is not the smallest, which indicates that the convergence speed of MSINGO is accelerated, but the memory used is increased.

## 5. MSINGO for Engineering Optimization Problems

This section verifies the efficiency of MSINGO in dealing with real-world optimization applications in six practical engineering design problems, including a tension/compression spring design problem (T/CSD) [68], cantilever beam design problem (CBD) [69], pressure vessel design problem (PVD) [70], welded beam design problem (WBD) [71], speed reducer design problem (SRD) [72], and three-bar truss design problem (T-bTD) [73]. Parameter settings of MSINGO and the other 11 comparative metaheuristic algorithms in this section are identical to those in Section 4.2.

### 5.1. Tension/Compression Spring Design Problem (T/CSD)

**Figure 13:**
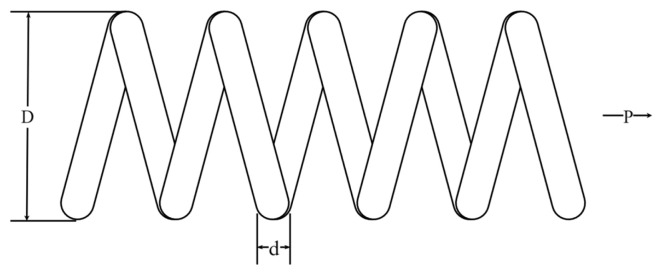
Schematic representation of T/CSD.

The tension/compression spring design (T/CSD) is an optimization problem to minimize the weight of a tension/compression spring with constraints. Its schematic diagram is shown in Figure 13. There are three variables that require optimization: spring wire diameter (d), spring coil diameter (D), and the number of active coils (P). The mathematical formula of T/CSD is as follows:

$$\mathrm{Consider}:\;\;\vec{x}=[x_{1}\;\;x_{2}\;\;x_{3}]=[d\;\;D\;\;P]$$

$$\mathrm{Minimize}:\;\;f(\vec{x})=(x_{3}+2)x_{2}x_{1}^{2}$$

$$\mathrm{Subject}\;\mathrm{to}:\;\;g_{1}(\vec{x})=1-\frac{x_{2}^{3}x_{3}}{71,785x_{1}^{4}}\leq 0$$

$$g_{2}(\vec{x})=\frac{4x_{2}^{2}-x_{1}x_{2}}{12,566(x_{2}x_{1}^{3}-x_{1}^{4})}+\frac{1}{5108x_{1}^{2}}-1\leq 0$$

$$g_{3}(\vec{x})=1-\frac{140.45x_{1}}{x_{2}^{2}x_{3}}\leq 0$$

$$g_{4}(\vec{x})=\frac{x_{1}+x_{2}}{1.5}-1\leq 0$$

$$\mathrm{Parameters}\;\mathrm{range}:\;\;0.05\leq x_{1}\leq 2,\;0.25\leq x_{2}\leq 1.3,\;2\leq x_{3}\leq 15$$

Table 16 presents the experimental results of MSINGO and 11 competitor algorithms in achieving the optimal solution for T/CSD. Based on these results, MSINGO ranks first among all the algorithms.

### 5.2. Cantilever Beam Design Problem (CBD)

**Figure 14:**
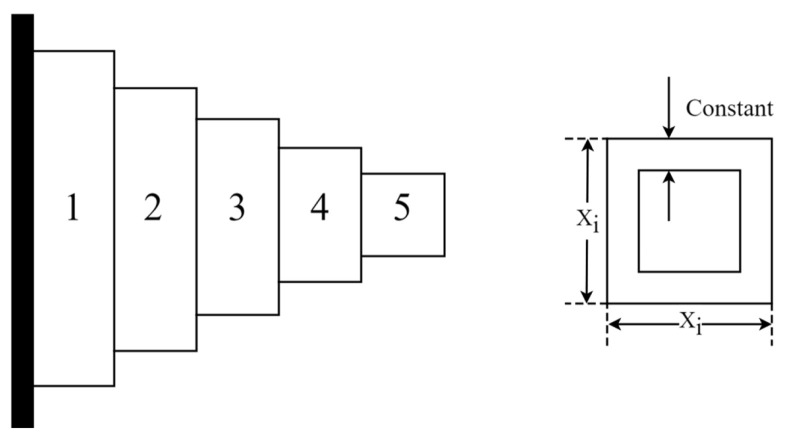
Schematic representation of CBD.

The cantilever beam design (CBD) is an engineering optimization problem to minimize the beam’s weight while meeting the constraint conditions. Schematic representation of the CBD is shown in Figure 14. A cantilever beam consists of five hollow blocks, each of which is a hollow square section with a constant thickness. There are five variables to be optimized. The optimization problem of CBD can be defined as follows:

$$\mathrm{Consider}:\;\;\vec{x}=[x_{1}\;x_{2}\;x_{3}\;x_{4}\;x_{5}]$$

$$\mathrm{Minimize}:\;\;f(\vec{x})=0.6224(x_{1}+x_{2}+x_{3}+x_{4}+x_{5})$$

$$\mathrm{Subject}\;\mathrm{to}:\;\;g(\vec{x})=\frac{61}{x_{1}^{3}}+\frac{37}{x_{2}^{3}}+\frac{19}{x_{3}^{3}}+\frac{7}{x_{4}^{3}}+\frac{1}{x_{5}^{3}}-1\leq 0$$

$$\mathrm{Parameter}\;\mathrm{range}:\;\;0.01\leq x_{1},x_{2},x_{3},x_{4},x_{5}\leq 100$$

Table 17 presents the optimal results. Based on Table 17, MSINGO outperforms all the 11 competitor algorithms.

### 5.3. Pressure Vessel Design Problem (PVD)

**Figure 15:**
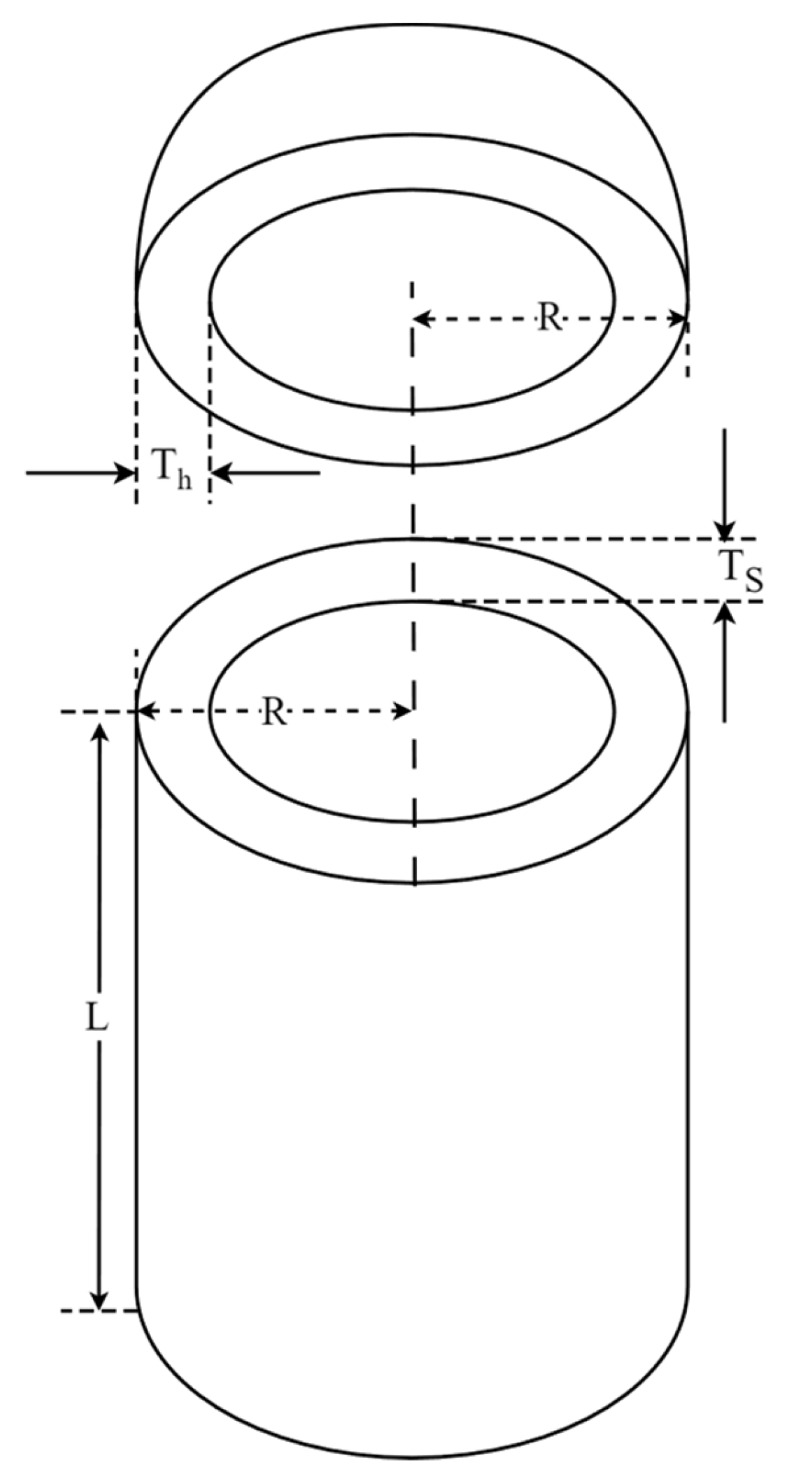
Schematic representation of PVD.

The pressure vessel design (PVD) problem aims at minimizing total vessel cost while satisfying the constraint conditions, as illustrated in Figure 15. This problem contains four optimization variables: thickness of the shell ($T_{S}$), thickness of the head ($T_{h}$), inner radius (R), and length of the cylindrical section excluding the head (L). The mathematical formula of the PVD optimization problem is as follows:

$$\mathrm{Consider}:\;\;\vec{x}=[x_{1}\;x_{2}\;x_{3}\;x_{4}]=[T_{s}\;T_{h}\;R\;L]$$

$$\mathrm{Minimize}:\;\;f(\vec{x})=0.6224x_{1}x_{3}x_{4}+1.7781x_{2}x_{3}^{2}+3.1661x_{1}^{2}x_{4}+19.84x_{1}^{2}x_{3}$$

$$\mathrm{Subject}\;\mathrm{to}:\;\;g_{1}(\vec{x})=-x_{1}+0.0193x_{3}\leq 0$$

$$g_{2}(\vec{x})=-x_{2}+0.00954x_{3}\leq 0$$

$$g_{3}(\vec{x})=-\pi x_{3}^{2}x_{4}-\frac{4}{3}\pi x_{3}^{3}+1,296,000\leq 0$$

$$g_{4}(\vec{x})=x_{4}-240\leq 0$$

$$\mathrm{Parameters}\;\mathrm{range}:\;\;0\leq x_{1},x_{2}\leq 99,\;\;\;10\leq x_{3},x_{4}\leq 200$$

Table 18 lists the optimal results of MSINGO and the competitor algorithms. MSINGO ranks second in solving PVD problems, just inferior to DE.

### 5.4. Welded Beam Design Problem (WBD)

**Figure 16:**
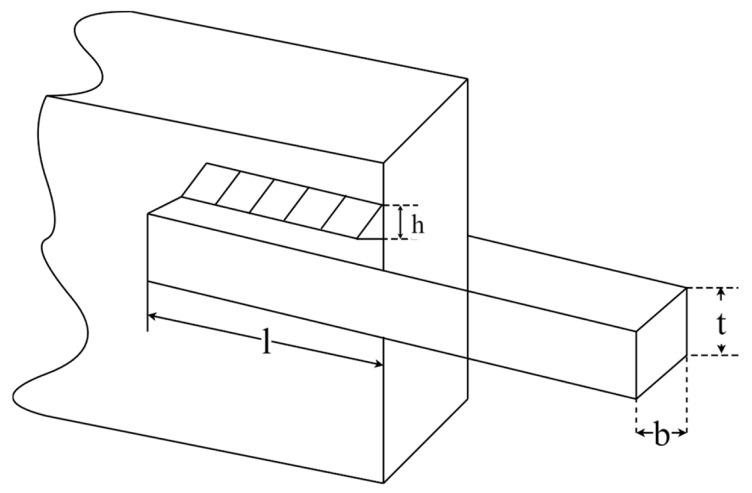
Schematic representation of WBD.

The welded beam design (WBD) problem is to minimize the cost of a welded beam. Figure 16 shows schematic representation of WBD, which has four optimization variables, including weld thickness (h), clamping bar length (l), bar height (t), and bar thickness (b). As seen in Figure 10. The mathematical formula of the WBD optimization problem is as follows:

$$\mathrm{Consider}:\;\;\vec{x}=[x_{1}\;x_{2}\;x_{3}\;x_{4}]=[h\;l\;t\;b]$$

$$\mathrm{Minimize}:\;\;f(\vec{x})=1.10471x_{1}^{2}x_{2}+0.04811x_{3}x_{4}(14.0+x_{2})$$

$$\mathrm{Subject}\;\mathrm{to}:\;\;g_{1}(\vec{x})=\tau (\vec{x})-\tau_{\max}\leq 0$$

$$g_{2}(\vec{x})=\sigma (\vec{x})-\sigma_{\max}\leq 0$$

$$g_{3}(\vec{x})=\delta (\vec{x})-\delta_{\max}\leq 0$$

$$g_{4}(\vec{x})=x_{1}-x_{4}\leq 0$$

$$g_{5}(\vec{x})=P-P_{c}(\vec{x})\leq 0$$

$$g_{6}(\vec{x})=0.125-x_{1}\leq 0$$

$$g_{7}(\vec{x})=1.10471x_{1}^{2}+0.04811x_{3}x_{4}(14.0+x_{2})-5.0\leq 0$$

$$\mathrm{Parameter}\;\mathrm{range}:\;\;0.1\leq x_{1},x_{4}\leq 2,\;\;\;0.1\leq x_{2,}x_{3}\leq 10$$

$$\mathrm{Where}:\;\;\tau (\vec{x})=\sqrt{{\left(\tau^{\prime }\right)}^{2}+2\tau^{\prime }\tau^{\prime \prime }\frac{x_{2}}{2R}+{\left(\tau^{\prime \prime }\right)}^{2}}$$

$$\tau^{\prime }=\frac{P}{\sqrt{2}x_{1}x_{2}},\;\;\;\tau^{\prime \prime }=\frac{MR}{J}$$

$$M=P(L+\frac{x_{2}}{2})$$

$$R=\sqrt{\frac{x_{2}^{2}}{4}+{\left(\frac{x_{1}+x_{3}}{2}\right)}^{2}}$$

$$J=2\left\{\sqrt{2}x_{1}x_{2}[\frac{x_{2}^{2}}{4}+{\left(\frac{x_{1}+x_{3}}{2}\right)}^{2}]\right\}$$

$$\sigma (\vec{x})=\frac{6PL}{x_{4}x_{3}^{2}},\;\;\delta (\vec{x})=\frac{6PL^{3}}{Ex_{3}^{2}x_{4}}$$

$$P_{c}(\vec{x})=\frac{4.013E\sqrt{\frac{x_{3}^{2}x_{4}^{6}}{36}}}{L^{2}}(1-\frac{x_{3}}{2L}\sqrt{\frac{E}{4G}})$$

$$P=6000\;\mathrm{lb},\;\;\;L=14\;\mathrm{in},\;\;\;\delta_{\max}=0.25\;\mathrm{in}$$

$$E=30\times 1^{6}\;\mathrm{psi},\;\;G=12\times 10^{6}\;\mathrm{psi}$$

$$\tau_{\max}=13,600\;\mathrm{psi},\;\sigma_{\max}=30,000\;\mathrm{psi}$$

The results of the WBD problem solved by MSINGO and compared algorithms are summarized in Table 19, where MSINGO ranks the first.

### 5.5. Speed Reducer Design Problem (SRD)

**Figure 17:**
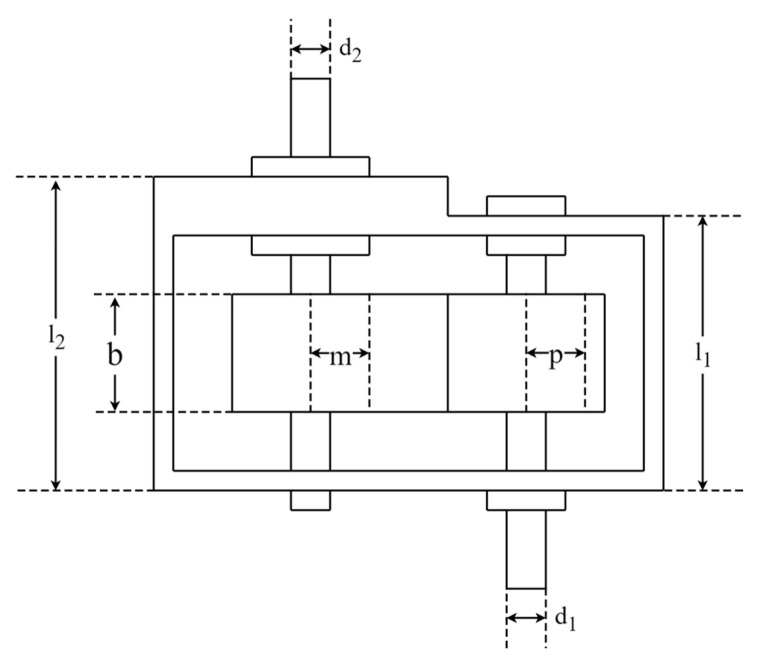
Schematic representation of SRD.

The speed reducer design (SRD) problem is an engineering optimization problem to minimize the weight of the reducer with constraints. A schematic representation of SRD is shown in Figure 17. It includes seven optimization variables: face width (b), module of teeth (m), pinion teeth count (p), length of the first shaft between bearings ($l_{1}$), length of the second shaft between bearings ($l_{2}$), diameter of the first shaft ($d_{1}$), and diameter of the second shaft ($d_{2}$). The mathematical formula of the SRD optimization problem is as follows:

$$\mathrm{Consider}:\;\;\vec{x}=[x_{1}\;\;\;x_{2}\;\;\;x_{3}\;\;\;x_{4}\;\;\;x_{5}\;\;\;x_{6}\;\;\;x_{7}]=[b\;\;m\;p\;l_{1}\;l_{2}\;d_{1}\;d_{2}]$$

$$\mathrm{Minimize}:\;\;\begin{array}{l} f(\vec{x})=0.7854x_{1}x_{2}^{2}(3.3333x_{3}^{2}+14.9334x_{3}-43.0934)-\\ \;\;\;1.508x_{1}(x_{6}^{2}+x_{7}^{2})+7.4777(x_{6}^{3}+x_{7}^{3})+0.7854(x_{4}x_{6}^{2}+x_{5}x_{7}^{2}) \end{array}$$

$$\mathrm{Subject}\;\mathrm{to}:\;\;g_{1}(\vec{x})=\frac{27}{x_{1}x_{2}^{2}x_{3}}-1\leq 0$$

$$g_{2}(\vec{x})=\frac{397.5}{x_{1}x_{2}^{2}x_{3}^{2}}-1\leq 0$$

$$g_{3}(\vec{x})=\frac{1.93x_{4}^{3}}{x_{2}x_{3}x_{6}^{4}}-1\leq 0$$

$$g_{4}(\vec{x})=\frac{1.93x_{5}^{3}}{x_{2}x_{3}x_{7}^{4}}-1\leq 0$$

$$g_{5}(\vec{x})=\frac{\sqrt{{\left(\frac{745x_{4}}{x_{2}x_{3}}\right)}^{2}+16.9\times 10^{6}}}{110.0x_{6}^{3}}-1\leq 0$$

$$g_{6}(\vec{x})=\frac{\sqrt{{\left(\frac{754x_{5}}{x_{2}x_{3}}\right)}^{2}+157.5\times 10^{6}}}{85.0x_{7}^{3}}-1\leq 0$$

$$g_{7}(\vec{x})=\frac{x_{2}x_{3}}{40}-1\leq 0$$

$$g_{8}(\vec{x})=\frac{5x_{2}}{x_{1}}-1\leq 0$$

$$g_{9}(\vec{x})=\frac{x_{1}}{12x_{2}}-1\leq 0$$

$$g_{10}(\vec{x})=\frac{1.5x_{6}+1.9}{x_{4}}-1\leq 0$$

$$g_{11}(\vec{x})=\frac{1.1x_{7}+1.9}{x_{5}}-1\leq 0$$

$$\mathrm{Parameter}\;\mathrm{range}:\;\;2.6\leq x_{1}\leq 3.6,\;\;\;\;0.7\leq x_{2}\leq 0.8,\;\;\;\;17.0\leq x_{3}\leq 28.0$$

$$7.3\leq x_{4}\leq 8.3,\;\;\;7.3\leq x_{5}\leq 8.3,\;\;\;2.9\leq x_{6}\leq 3.9$$

$$5.0\leq x_{7}\leq 5.5$$

Table 20 displays the optimization results for SRD. MSINGO ranks first along with NGO, DE, and BWO.

### 5.6. Three-Bar Truss Design Problem (T-bTD)

**Figure 18:**
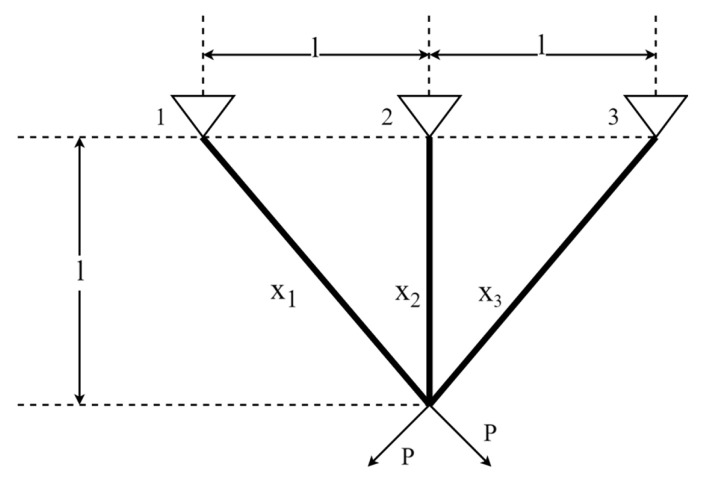
Schematic representation of T-bTD.

The three-bar truss design (T-bTD) problem is an optimization problem in civil engineering where the objective is to minimize the volume of the three-bar truss. The schematic representation of T-bTD is shown in Figure 18. It has two optimization variables, namely $x_{1}$ and $x_{2}$. The mathematical formula of T-bTD is defined as follows:

$$\mathrm{Consider}:\;\;\vec{x}=[x_{1}\;x_{2}]$$

$$\mathrm{Minimize}:\;\;f(\vec{x})=(2\sqrt{2}x_{1}+x_{2})l$$

$$\mathrm{Subject}\;\mathrm{to}:\;\;g_{1}(\vec{x})=\frac{\sqrt{2}x_{1}+x_{2}}{\sqrt{2}x_{1}^{2}+2x_{1}x_{2}}P-\sigma \leq 0$$

$$g_{2}(\vec{x})=\frac{x_{2}}{\sqrt{2}x_{1}^{2}+2x_{1}x_{2}}P-\sigma \leq 0$$

$$g_{3}(\vec{x})=\frac{1}{\sqrt{2}x_{2}+x_{1}}P-\sigma \leq 0$$

$$\mathrm{Parameter}\;\mathrm{range}:\;\;0\leq x_{1},x_{2}\leq 1$$

$$\mathrm{Where}:\;\;l=100\;\mathrm{cm},P=2\;\mathrm{kN}/{\mathrm{cm}}^{2},\sigma =\mathrm{kN}/{\mathrm{cm}}^{2}$$

Table 21 presents the experimental results. MSINGO ranks second, behind DE.

**Figure 19:**
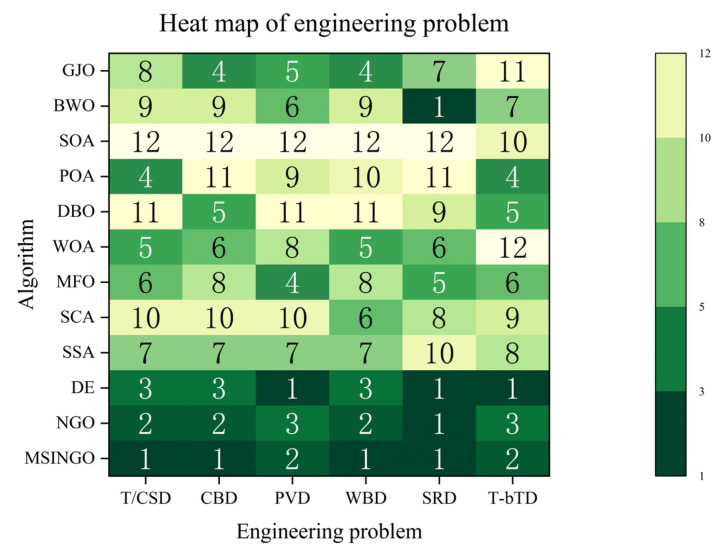
The heat map of different algorithms on 6 engineering problems.

Figure 19 shows a heat map of 12 algorithms on six engineering applications. MSINGO ranks the lowest overall, proving that MSINGO has excellent performance in solving engineering problems.

Different engineering applications have different conditions, so one algorithm cannot be applicable to all engineering applications. In most cases, MSINGO outperforms other algorithms, but performs worse than DE in the pressure vessel design problem (PVD) and the three-bar truss design problem (T-bTD). Therefore, for different practical problems, algorithm improvement also requires targeted analysis.

## 6. Conclusions and Future Work

In this paper, we propose a multi-strategy improved northern goshawk optimization named MSINGO. Firstly, cubic mapping is applied in the population initialization to improve the population diversity of the algorithm. Secondly, weighted stochastic difference mutation is added in the exploration phase to make it jump out of the local optimal solution. Finally, we use the weighted sine and cosine optimization strategy instead of the original exploitation formula to enhance convergence speed of the MSINGO. We analyzed the impact of the three strategies on NGOs and verified the effectiveness of the proposed algorithm. Then, MSINGO is compared with 11 well-known algorithms on CEC2017 test functions. Comparative experiments include comparison of exploitation ability, exploration ability, local optimal avoidance ability, and scalability. The comparative experimental results show that in the vast majority of cases, MSINGO’s exploitation ability, exploration ability, local optimal avoidance ability, and scalability are superior to those of competitive algorithms. Additionally, the implementation of MSINGO in addressing six engineering design optimization issues demonstrated the high capability of MSINGO in real-world optimization problems.

In the future, we will verify our algorithm on large size datasets and study its performance on large size datasets, such as parameter selection for a neural network model and so on. We will also use hybrid methods to validate the performance of the algorithm, such as combining it with faster gradient-based methods.

## Figures and Tables

**Table 2:** Details of the CEC2017 benchmark functions.

Category	ID	Function	Range	$f_{min}$
Unimodal functions	C1	Shifted and Rotated Bent Cigar Function	[−100, 100]	100
	C2	Shifted and Rotated Zakharov Function	[−100, 100]	200
Multimodal functions	C3	Shifted and Rotated Rosenbrock’s Function	[−100, 100]	300
	C4	Shifted and Rotated Rastrigin’s Function	[−100, 100]	400
	C5	Shifted and Rotated Schaffer’s F7 Function	[−100, 100]	500
	C6	Shifted and Rotated Lunacek Bi-Rastrigin’s Function	[−100, 100]	600
	C7	Shifted and Rotated Non-Continuous Rastrigin’s Function	[−100, 100]	700
	C8	Shifted and Rotated Levy Function	[−100, 100]	800
	C9	Shifted and Rotated Schwefel’s Function	[−100, 100]	900
Hybrid functions	C10	Hybrid Function 1 (*N*= 3)	[−100, 100]	1000
	C11	Hybrid Function 2 (*N* = 3)	[−100, 100]	1100
	C12	Hybrid Function 3 (*N* = 3)	[−100, 100]	1200
	C13	Hybrid Function 4 (*N* = 4)	[−100, 100]	1300
	C14	Hybrid Function 5 (*N* = 4)	[−100, 100]	1400
	C15	Hybrid Function 6 (*N* = 4)	[−100, 100]	1500
	C16	Hybrid Function 7 (*N* = 5)	[−100, 100]	1600
	C17	Hybrid Function 8 (*N* = 5)	[−100, 100]	1700
	C18	Hybrid Function 9 (*N* = 5)	[−100, 100]	1800
	C19	Hybrid Function 10 (*N* = 6)	[−100, 100]	1900
Composition functions	C20	Composition Function 1 (*N* = 3)	[−100, 100]	2000
	C21	Composition Function 2 (*N* = 3)	[−100, 100]	2100
	C22	Composition Function 3 (*N* = 4)	[−100, 100]	2200
	C23	Composition Function 4 (*N* = 4)	[−100, 100]	2300
	C24	Composition Function 5 (*N* = 5)	[−100, 100]	2400
	C25	Composition Function 6 (*N* = 5)	[−100, 100]	2500
	C26	Composition Function 7 (*N* = 6)	[−100, 100]	2600
	C27	Composition Function 8 (*N* = 6)	[−100, 100]	2700
	C28	Composition Function 9 (*N* = 3)	[−100, 100]	2800
	C29	Composition Function 10 (*N* = 3)	[−100, 100]	2900

**Table 3:** Parameter settings of the competitors and proposed MSINGO.

Category	Algorithms	Name of the Parameter	Value of the Parameter
Highly cited	DE	PCr,F	0.8, 0.85
	MFO	b	1
	WOA	a,a2,b	[0, 2], [−2, −1], 1
	SCA	a	2
	SOA	$f_{c}$	2
Recently proposed	SSA	ST,PD,SD	0.6, 0.7, 0.2
	DBO	PballRolling,PbroodBall,PSmall,Pthief, b,k,S	0.2, 0.4, 0.2, 0.4, b = 0.3, k = 0.1, S = 0.5
	POA	R	0.2
	BWO	$w_{f}$	[0.1, 0.05]
	GJO	β	0.5
	NGO	R	[0, 0.02]
Our proposed	MSINGO	a,β	2.595, 1.5

**Table 4:** Various NGO algorithms from three mechanisms.

	Cubic Mapping (C)	Weighted Stochastic Difference Variation (WS)	Weighted Sine and Cosine Optimization (WSC)
NGO	0	0	0
C_NGO	1	0	0
WS_NGO	0	1	0
WSC_NGO	0	0	1
C_WS_NGO	1	1	0
C_WSC_NGO	1	0	1
WS_WSC_NGO	0	1	1
MSINGO	1	1	1

**Table 5:** Experimental results of strategy comparison on CEC2017 test functions.

ID		NGO	C_NGO	WS_NGO	WSC_NGO	C_WS_NGO	C_WSC_NGO	WS_WSC_NGO	MSINGO
C1	Ave	1.8025 × 10^7^	1.4315 × 10^8^	5.5679 × 10^3^	3.8139 × 10^6^	**5.3757 × 10** ^3^	4.0069 × 10^6^	8.2264 × 10^3^	1.0583 × 10^4^
	Std	1.9969 × 10^7^	1.8369 × 10^8^	4.6062 × 10^3^	1.4174 × 10^6^	**5.1807 × 10** ^3^	1.7799 × 10^6^	6.2044 × 10^3^	7.6418 × 10
C2	Ave	9.5971 × 10^2^	2.2737 × 10^3^	2.0586 × 10^2^	3.3318 × 10^2^	**2.0162 × 10** ^2^	3.2309 × 10^2^	2.1121 × 10^2^	2.0902 × 10^2^
	Std	4.8997 × 10^2^	8.6804 × 10^2^	1.3416 × 10^1^	5.7545 × 10^1^	**1.9350**	5.5625 × 10^1^	7.6682	5.5120
C3	Ave	4.2824 × 10^2^	4.7716 × 10^2^	3.5739 × 10^2^	3.9802 × 10^2^	3.7833 × 10^2^	3.9686 × 10^2^	3.5959 × 10^2^	**3.5318 × 10** ^2^
	Std	4.6788 × 10^1^	5.3148 × 10^1^	2.8096 × 10^1^	3.1249 × 10^1^	4.3003 × 10^1^	4.4813 × 10^1^	2.9606 × 10^1^	**2.6328 × 10** ^1^
C4	Ave	1.0209 × 10^3^	1.2609 × 10^3^	**5.7725 × 10** ^2^	7.0194 × 10^2^	5.8034 × 10^2^	7.0097 × 10^2^	6.3334 × 10^2^	6.2263 × 10^2^
	Std	2.1185 × 10^2^	5.0975 × 10^2^	**3.1176 × 10** ^1^	5.1463 × 10^1^	2.9463 × 10^1^	3.8466 × 10^1^	3.0272 × 10^1^	3.0987 × 10^1^
C5	Ave	5.0000 × 10^2^	5.0000 × 10^2^	5.0000 × 10^2^	**5.0000 × 10** ^2^	5.0000 × 10^2^	5.0000 × 10^2^	5.0000 × 10^2^	5.0000 × 10^2^
	Std	1.1436 × 10^−3^	2.5502 × 10^−3^	8.8501 × 10^−4^	**3.6425 × 10** ^−4^	6.3949 × 10^−4^	3.7749 × 10^−4^	4.5111 × 10^−4^	4.1208 × 10^−4^
C6	Ave	3.4794 × 10^3^	1.9116 × 10^4^	3.2849 × 10^3^	1.5336 × 10^4^	**2.6826 × 10** ^3^	1.4086 × 10^4^	8.7875 × 10^3^	8.4491 × 10^3^
	Std	1.3695 × 10^3^	6.3324 × 10^3^	1.2963 × 10^3^	6.6900 × 10^3^	**7.8237 × 10** ^2^	5.6652 × 10^3^	3.6049 × 10^3^	3.9353 × 10^3^
C7	Ave	7.0013 × 10^2^	7.0103 × 10^2^	**7.0010 × 10** ^2^	7.0022 × 10^2^	7.0011 × 10^2^	7.0020 × 10^2^	7.0014 × 10^2^	7.0015 × 10^2^
	Std	6.0608 × 10^−2^	2.6993 × 10^−1^	**7.1534 × 10** ^−2^	8.5846 × 10^−2^	4.2965 × 10^−2^	7.3934 × 10^−2^	6.2216 × 10^−2^	6.1209 × 10^−2^
C8	Ave	8.0554 × 10^2^	8.0587 × 10^2^	**8.0154 × 10** ^2^	8.0425 × 10^2^	8.0158 × 10^2^	8.0471 × 10^2^	8.0199 × 10^2^	8.0156 × 10^2^
	Std	1.9518	1.9525	**1.4377**	1.4377	1.22370	1.5104	1.3990	1.0497
C9	Ave	5.1677 × 10^3^	7.7212 × 10^3^	5.2430 × 10^3^	5.2093 × 10^3^	5.2409 × 10^3^	5.1227 × 10^3^	5.2556 × 10^3^	**5.0619 × 10** ^3^
	Std	4.5304 × 10^2^	3.6230 × 10^2^	4.0000 × 10^2^	5.7974 × 10^2^	4.0613 × 10^2^	7.1581 × 10^2^	4.8143 × 10^2^	**5.4751 × 10** ^2^
C10	Ave	3.5772 × 10^4^	2.9359 × 10^4^	3.8583 × 10^4^	**1.2361 × 10** ^4^	2.5199 × 10^4^	1.3668 × 10^4^	2.2557 × 10^4^	1.6519 × 10^4^
	Std	2.3458 × 10^4^	1.6215 × 10^4^	2.7401 × 10^4^	**6.2903 × 10** ^3^	1.5051 × 10^4^	6.8203 × 10^3^	2.0052 × 10^4^	8.7681 × 10^3^
C11	Ave	1.9569 × 10^5^	3.9197 × 10^5^	1.0143 × 10^5^	2.6041 × 10^5^	1.1863 × 10^5^	2.9005 × 10^5^	9.7033 × 10^5^	**8.1669 × 10** ^4^
	Std	1.9607 × 10^5^	3.3473 × 10^5^	8.6213 × 10^4^	2.3087 × 10^5^	1.0390 × 10^5^	3.2213 × 10^5^	8.3390 × 10^4^	**5.8853 × 10** ^4^
C12	Ave	2.5452 × 10^4^	2.3404 × 10^4^	**5.7697 × 10** ^3^	1.0315 × 10^5^	6.2603 × 10^3^	4.9563 × 10^4^	6.5499 × 10^3^	7.6979 × 10^3^
	Std	2.6804 × 10^4^	1.2787 × 10^4^	**4.0495 × 10** ^3^	1.1173 × 10^5^	5.3015 × 10^3^	6.1465 × 10^4^	5.3117 × 10^3^	5.5292 × 10^3^
C13	Ave	2.5841 × 10^5^	3.1302 × 10^5^	**1.1252 × 10** ^5^	3.1334 × 10^5^	1.1695 × 10^5^	2.5456 × 10^5^	1.5680 × 10^5^	1.5022 × 10^5^
	Std	1.4319 × 10^5^	1.3164 × 10^5^	**3.6354 × 10** ^4^	1.1721 × 10^5^	3.9481 × 10^4^	1.0037 × 10^5^	8.8740 × 10^4^	4.4144 × 10^4^
C14	Ave	2.6773 × 10^4^	2.4840 × 10^4^	2.1099 × 10^4^	2.7076 × 10^4^	2.0208 × 10^4^	2.2563 × 10^4^	1.6073 × 10^4^	**1.3331 × 10** ^4^
	Std	1.0410 × 10^4^	1.0861 × 10^4^	1.0634 × 10^4^	1.0504 × 10^4^	1.4964 × 10^4^	8.0369 × 10^3^	5.6588 × 10^3^	**7.8585 × 10** ^3^
C15	Ave	1.9647 × 10^3^	6.3238 × 10^3^	1.8444 × 10^3^	2.3482 × 10^3^	**1.7647 × 10** ^3^	3.3227 × 10^3^	2.1792 × 10^3^	1.9586 × 10^3^
	Std	3.4647 × 10^2^	6.1986 × 10^3^	3.5904 × 10^2^	8.2637 × 10^2^	**2.5106 × 10** ^2^	1.4183 × 10^3^	1.2759 × 10^3^	3.6559 × 10^2^
C16	Ave	7.3627 × 10^3^	4.5647 × 10^3^	7.2881 × 10^3^	**3.3705 × 10** ^3^	6.4137 × 10^3^	3.4096 × 10^3^	5.0775 × 10^3^	4.4896 × 10^3^
	Std	7.1224 × 10^3^	3.0930 × 10^3^	6.9245 × 10^3^	**2.2489 × 10** ^3^	3.8862 × 10^3^	1.6588 × 10^3^	3.2278 × 10^3^	2.6157 × 10^3^
C17	Ave	9.2979 × 10^4^	1.0940 × 10^5^	**4.7520 × 10** ^4^	1.0834 × 10^5^	5.0553 × 10^4^	1.1181 × 10^5^	7.8437 × 10^4^	8.5337 × 10^4^
	Std	4.5633 × 10^4^	3.8472 × 10^4^	**1.6387 × 10** ^4^	2.7706 × 10^4^	1.6759 × 10^4^	3.6410 × 10^4^	2.1293 × 10^4^	3.0722 × 10^4^
C18	Ave	1.7265 × 10^4^	**8.2445 × 10** ^3^	2.0264 × 10^4^	1.5480 × 10^4^	3.7698 × 10^4^	1.3146 × 10^4^	2.5454 × 10^4^	2.2163 × 10^4^
	Std	1.7684 × 10^4^	**1.0789 × 10** ^4^	1.7719 × 10^4^	1.7940 × 10^4^	2.6475 × 10^4^	1.5760 × 10^4^	2.1266 × 10^4^	1.7829 × 10^4^
C19	Ave	2.7377 × 10^3^	2.7541 × 10^3^	2.4082 × 10^3^	2.3599 × 10^3^	2.3419 × 10^3^	2.3971 × 10^3^	2.2661 × 10^3^	**2.2469 × 10** ^3^
	Std	2.7274 × 10^2^	3.4635 × 10^2^	2.7271 × 10^2^	1.9647 × 10^2^	2.8974 × 10^2^	2.3299 × 10^2^	1.4353 × 10^2^	**1.6479 × 10** ^2^
C20	Ave	2.6466 × 10^3^	2.6348 × 10^3^	**2.2183 × 10** ^3^	2.3056 × 10^3^	2.2987 × 10^3^	2.3848 × 10^3^	2.2597 × 10^3^	2.3231 × 10^3^
	Std	4.8847 × 10^2^	3.3976 × 10^2^	**1.3459 × 10** ^2^	1.6827 × 10^2^	1.3206 × 10^2^	1.5305 × 10^2^	1.6844 × 10^2^	1.5802 × 10^2^
C21	Ave	2.3416 × 10^3^	2.3513 × 10^3^	2.2794 × 10^3^	2.2876 × 10^3^	2.2796 × 10^3^	**2.2742 × 10** ^3^	2.2998 × 10^3^	2.2752 × 10^3^
	Std	2.7963 × 101	4.3453 × 10^1^	6.6238	7.5533	7.8133	**6.5709**	1.1813 × 10^1^	6.6923
C22	Ave	3.0030 × 10^3^	3.4859 × 10^3^	2.4293 × 10^3^	2.6507 × 10^3^	**2.4026 × 10** ^3^	2.6507 × 10^3^	2.4386 × 10^3^	2.4422 × 10^3^
	Std	5.7231 × 10^2^	3.9136 × 10^2^	8.0678 × 10^1^	1.1595 × 10^2^	**8.2796 × 10** ^−1^	1.1331 × 10^2^	9.8880 × 10^1^	1.1063 × 10^2^
C23	Ave	2.8273 × 10^3^	3.2852 × 10^3^	2.5014 × 10^3^	2.6288 × 10^3^	**2.5012 × 10** ^3^	2.6432 × 10^3^	2.5352 × 10^3^	2.5146 × 10^3^
	Std	4.6000 × 10^2^	5.5984 × 10^2^	6.0767 × 10^−1^	2.8852 × 10^1^	**5.1043 × 10** ^−1^	6.9075 × 10^1^	9.7281 × 10^1^	6.2048 × 10^1^
C24	Ave	2.8767 × 10^3^	2.8928 × 10^3^	2.8249 × 10^3^	2.8460 × 10^3^	2.8281 × 10^3^	2.8510 × 10^3^	2.8305 × 10^3^	**2.8249 × 10** ^3^
	Std	2.2767 × 10^1^	3.8269 × 10^1^	7.6830	1.3856 × 10^1^	8.9261	1.5720 × 10^1^	9.3798	**4.6406**
C25	Ave	3.3543E × 10^3^	3.3604 × 10^3^	3.3580 × 10^3^	3.3287 × 10^3^	3.3595 × 10^3^	**3.3276 × 10** ^3^	3.3310 × 10^3^	3.3352 × 10^3^
	Std	1.8431 × 10^1^	2.2434 × 10^1^	1.8335 × 10^1^	2.0772	1.9466 × 10^1^	**9.0204**	4.4104	1.3340 × 10^1^
C26	Ave	3.1376 × 10^3^	3.1378 × 10^3^	3.1446 × 10^3^	**3.1253 × 10** ^3^	3.1450 × 10^3^	3.1339 × 10^3^	3.1338 × 10^3^	3.1284 × 10^3^
	Std	2.1172 × 10^1^	1.8583 × 10^1^	1.8694 × 10^1^	**1.8782 × 10** ^1^	2.5270 × 10^1^	2.5982 × 10^1^	2.7872 × 10^1^	1.9031 × 10^1^
C27	Ave	2.9989 × 10^3^	3.0790 × 10^3^	2.9391 × 10^3^	2.7340 × 10^3^	2.8915 × 10^3^	2.7611 × 10^3^	2.7663 × 10^3^	**2.7338 × 10** ^3^
	Std	1.3136 × 10^2^	8.9258 × 10^1^	1.5966 × 10^2^	5.3534 × 10^1^	1.7577 × 10^2^	1.3843 × 10^2^	1.1840 × 10^2^	**4.6613 × 10** ^1^
C28	Ave	3.8901 × 10^4^	3.9198 × 10^4^	2.7089 × 10^4^	2.3630 × 10^4^	2.8494 × 10^4^	3.7390 × 10^4^	2.1817 × 10^4^	**1.7619 × 10** ^4^
	Std	3.8092 × 10^4^	3.4223 × 10^4^	2.1814 × 10^4^	2.2986 × 10^4^	1.6735 × 10^4^	9.4507 × 10^4^	1.6813 × 10^4^	**1.5936 × 10** ^4^
C29	Ave	7.7663 × 10^4^	4.4846 × 10^5^	4.6742 × 10^5^	6.5721 × 10^4^	1.0671 × 10^5^	4.1435 × 10^4^	**2.5975 × 10** ^4^	4.2697 × 10^4^
	Std	7.1995 × 10^4^	7.1173 × 10^5^	6.3771 × 10^5^	5.3622 × 10^4^	1.9750 × 10^5^	1.8536 × 10^4^	**1.1253 × 10** ^4^	6.2395 × 10^4^

Bold is the best result of all algorithms.

**Table 6:** Friedman test results of different strategies.

	Overall Rank	Average Rank	+/−/=
NGO	7	6.034	26/3/0
C_NGO	8	7.276	28/1/0
WS_NGO	2	3.449	15/14/0
WSC_NGO	6	4.759	22/7/0
C_WS_NGO	3	3.552	17/12/0
C_WSC_NGO	5	4.552	23/6/0
WS_WSC_NGO	4	3.621	20/9/0
MSINGO	1	2.793	~

**Table 7:** Comparison results on unimodal functions (C1–C2, dimension = 30).

		DE	SSA	SCA	MFO	WOA	DBO
C1	Avg	7.1157 × 10^9^	1.8983 × 10^10^	2.1073 × 10^10^	2.0367 × 10^10^	1.6594 × 10^10^	6.5809 × 10^9^
	Std	1.7525 × 10^9^	4.9407 × 10^9^	4.1159 × 10^9^	1.5291 × 10^10^	3.1904 × 10^9^	4.6532 × 10^9^
	Rank	4	8	10	9	7	3
C2	Avg	5.2832 × 10^4^	5.5643 × 10^4^	4.5035 × 10^4^	6.5401 × 10^4^	5.2580 × 10^4^	5.0283 × 10^4^
	Std	9.5379 × 10^3^	1.3621 × 10^4^	9.9155 × 10^3^	2.8648 × 10^4^	9.9181 × 10^3^	2.0199 × 10^4^
	Rank	7	6	3	10	5	4
		**POA**	**SOA**	**BWO**	**GJO**	**NGO**	**MSINGO**
C1	Avg	6.2347 × 10^10^	5.4888 × 10^10^	7.5221 × 10^9^	1.3628 × 10^10^	1.8025 × 10^7^	**1.0583 × 10** ^4^
	Std	5.2900 × 10^9^	5.5611 × 10^9^	1.8633 × 10^9^	2.3413 × 10^9^	1.9969 × 10^7^	**7.6416 × 10** ^3^
	Rank	12	11	5	6	2	**1**
C2	Avg	1.2412 × 10^5^	1.1839 × 10^5^	5.5750 × 10^4^	5.6468 × 10^4^	9.5971 × 10^2^	**2.0902 × 10** ^2^
	Std	3.7176 × 10^4^	2.2784 × 10^4^	1.1262 × 10^4^	7.0274 × 10^3^	4.8997 × 10^2^	**5.5120**
	Rank	12	11	8	9	2	**1**

Bold is the best result of all algorithms.

**Table 8:** Results for *p*-value from Wilcoxon signed-rank test on unimodal functions (C1–C2, dimension = 30).

Ours vs.	DE	SSA	SCA	MFO	WOA	DBO	POA	SOA	BWO	GJO	NGO
C1	2.87 × 10^−11^	2.87 × 10^−11^	2.87 × 10^−11^	2.87 × 10^−11^	2.87 × 10^−11^	2.87 × 10^−11^	2.87 × 10^−11^	2.87 × 10^−11^	2.87 × 10^−11^	2.87 × 10^−11^	2.87 × 10^−11^
C2	2.87 × 10^−11^	2.87 × 10^−11^	2.87 × 10^−11^	2.87 × 10^−11^	2.87 × 10^−11^	2.87 × 10^−11^	2.87 × 10^−11^	2.87 × 10^−11^	2.87 × 10^−11^	2.87 × 10^−11^	2.87 × 10^−11^

**Table 9:** Comparison results on multimodal functions (C3–C9, dimension = 30).

		DE	SSA	SCA	MFO	WOA	DBO
C3	Avg	1.0418 × 10^3^	2.9128 × 10^3^	2.9573 × 10^3^	4.2844 × 10^3^	1.7357 × 10^3^	1.3672 × 10^3^
	Std	1.8976 × 10^2^	1.1603 × 10^3^	9.5803 × 10^2^	3.0445 × 10^3^	6.9362 × 10^2^	9.3724 × 10^2^
	Rank	3	8	9	10	7	6
C4	Avg	6.9329 × 10^3^	2.7862 × 10^4^	2.5376 × 10^4^	2.7085 × 10^4^	1.7964 × 10^4^	1.9735 × 10^4^
	Std	1.2039 × 10^3^	7.4911 × 10^3^	5.2626 × 10^3^	1.4029 × 10^4^	6.8852 × 10^3^	1.1157 × 10^4^
	Rank	3	10	8	9	6	7
C5	Avg	5.0003 × 10^2^	5.0001 × 10^2^	5.0003 × 10^2^	5.0001 × 10^2^	5.0001 × 10^2^	5.0001 × 10^2^
	Std	4.9406 × 10^−3^	5.5137 × 10^−3^	9.6118 × 10^−3^	5.6648 × 10^−3^	4.7371 × 10^−3^	7.4612 × 10^−3^
	Rank	9	5	11	6	4	8
C6	Avg	4.5943 × 10^4^	1.5106 × 10^4^	5.3867 × 10^4^	1.0947 × 10^4^	3.3083 × 10^4^	3.9703 × 10^4^
	Std	9.3287 × 10^3^	4.8363 × 10^3^	1.6393 × 10^4^	7.4864 × 10^3^	8.3105 × 10^3^	1.5718 × 10^4^
	Rank	9	4	11	3	6	7
C7	Avg	7.0337 × 10^2^	7.0074 × 10^2^	7.0281 × 10^2^	7.0071 × 10^2^	7.0108 × 10^2^	7.0221 × 10^2^
	Std	8.1562 × 10^−1^	6.9344 × 10^−1^	7.3905 × 10^−1^	6.6394 × 10^−1^	4.6689 × 10^−1^	8.8467 × 10^−1^
	Rank	11	4	10	3	6	8
C8	Avg	8.1667 × 10^2^	8.1785 × 10^2^	8.1640 × 10^2^	8.2556 × 10^2^	8.1415 × 10^2^	8.1363 × 10^2^
	Std	4.2145	4.2574	4.3496	8.1639	6.1010	7.5496
	Rank	8	9	7	10	6	5
C9	Avg	8.5354 × 10^3^	6.0192 × 10^3^	8.6150 × 10^3^	5.6357 × 10^3^	6.9107 × 10^3^	7.6447 × 10^3^
	Std	3.5466 × 10^2^	6.8392 × 10^2^	2.4630 × 10^2^	8.1562 × 10^2^	5.5922 × 10^2^	6.0268 × 10^2^
	Rank	10	4	11	3	5	7
		**POA**	**SOA**	**BWO**	**GJO**	**NGO**	**MSINGO**
C3	Avg	1.7796 × 10^4^	1.5998 × 10^4^	1.1487 × 10^3^	1.3002 × 10^3^	4.2824 × 10^2^	**3.5319 × 10** ^2^
	Std	2.9417 × 10^3^	4.9858 × 10^3^	2.6341 × 10^2^	3.9099 × 10^2^	4.6788 × 10^1^	**2.6328 × 10** ^1^
	Rank	12	11	4	5	2	**1**
C4	Avg	8.7121 × 10^4^	7.2960 × 10^4^	9.1229 × 10^3^	1.1006 × 10^4^	1.0209 × 10^3^	**6.2263 × 10** ^2^
	Std	1.1259 × 10^4^	1.1609 × 10^4^	2.5040 × 10^3^	3.8440 × 10^3^	2.1185 × 10^2^	**3.0987 × 10** ^1^
	Rank	12	11	4	5	2	**1**
C5	Avg	5.0004 × 10^2^	5.0003 × 10^2^	5.0001 × 10^2^	5.0001 × 10^2^	5.0000 × 10^2^	**5.0000 × 10** ^2^
	Std	1.1975 × 10^−2^	8.2257 × 10^−3^	6.4051 × 10^−3^	3.9437 × 10^−3^	1.1436 × 10^−3^	**4.1208 × 10** ^−4^
	Rank	12	10	7	3	2	**1**
C6	Avg	7.5547 × 10^4^	5.3641 × 10^4^	4.3028 × 10^4^	2.5471 × 10^4^	**3.4794 × 10** ^3^	8.4491 × 10^3^
	Std	1.5711 × 10^4^	1.5298 × 10^4^	8.0718 × 10^3^	7.4351 × 10^3^	**1.3695 × 10** ^3^	3.9353 × 10^3^
	Rank	12	10	8	5	**1**	2
C7	Avg	7.0428 × 10^2^	7.0256 × 10^2^	7.0171 × 10^2^	7.0100 × 10^2^	**7.0013 × 10** ^2^	7.0015 × 10^2^
	Std	9.5099 × 10^−1^	8.0624 × 10^−1^	7.5813 × 10^−1^	3.4868 × 10^−1^	**6.0608 × 10** ^−2^	6.1209 × 10^−2^
	Rank	12	9	7	5	**1**	2
C8	Avg	8.3070 × 10^2^	8.3700 × 10^2^	8.0984 × 10^2^	8.0862 × 10^2^	8.0554 × 10^2^	**8.0156 × 10** ^2^
	Std	6.7501	8.5159	3.1223	2.1307	1.9519	**1.0497**
	Rank	11	12	4	3	2	**1**
C9	Avg	8.1098 × 10^3^	9.1949 × 10^3^	7.9685 × 10^3^	7.3134 × 10^3^	5.1677 × 10^3^	**5.0619 × 10** ^3^
	Std	3.7913 × 10^2^	6.8997 × 10^2^	4.3993 × 10^2^	4.2661 × 10^2^	4.5304 × 10^2^	**5.4751 × 10** ^2^
	Rank	9	12	8	6	2	**1**

Bold is the best result of all algorithms.

**Table 10:** Results for *p*-value from Wilcoxon signed-rank test on multimodal functions (C3-C9, dimension = 30).

Ours vs.	DE	SSA	SCA	MFO	WOA	DBO	POA	SOA	BWO	GJO	NGO
C3	2.87 × 10^−11^	2.87 × 10^−11^	2.87 × 10^−11^	2.87 × 10^−11^	2.87 × 10^−11^	2.87 × 10^−11^	2.87 × 10^−11^	2.87 × 10^−11^	2.87 × 10^−11^	2.87 × 10^−11^	2.87 × 10^−11^
C4	2.87 × 10^−11^	2.87 × 10^−11^	2.87 × 10^−11^	2.87 × 10^−11^	2.87 × 10^−11^	2.87 × 10^−11^	2.87 × 10^−11^	2.87 × 10^−11^	2.87 × 10^−11^	2.87 × 10^−11^	2.87 × 10^−11^
C5	2.87 × 10^−11^	3.66 × 10^−9^	2.87 × 10^−11^	2.49 × 10^−8^	2.87 × 10^−11^	2.87 × 10^−11^	2.87 × 10^−11^	2.87 × 10^−11^	2.87 × 10^−11^	2.87 × 10^−11^	6.80 × 10^−8^
C6	2.87 × 10^−11^	1.93 × 10^−6^	2.87 × 10^−11^	3.75 × 10^−1^	2.87 × 10^−11^	1.39 × 10^−10^	2.87 × 10^−11^	3.18 × 10^−11^	2.87 × 10^−11^	3.88 × 10^−11^	3.39 × 10^−7^
C7	2.87 × 10^−11^	8.51 × 10^−7^	2.87 × 10^−11^	3.21 × 10^−6^	2.87 × 10^−11^	2.87 × 10^−11^	2.87 × 10^−11^	2.87 × 10^−11^	3.16 × 10^−11^	2.87 × 10^−11^	2.04 × 10^−1^
C8	2.87 × 10^−11^	2.87 × 10^−11^	2.87 × 10^−11^	2.87 × 10^−11^	2.87 × 10^−11^	5.23 × 10^−11^	2.87 × 10^−11^	2.87 × 10^−11^	2.87 × 10^−11^	2.87 × 10^−11^	1.06 × 10^−8^
C9	2.87 × 10^−11^	7.32 × 10^−7^	2.87 × 10^−11^	1.56 × 10^−3^	4.29 × 10^−11^	2.87 × 10^−11^	2.87 × 10^−11^	2.87 × 10^−11^	2.87 × 10^−11^	3.18 × 10^−11^	4.25 × 10^−1^

**Table 11:** Comparison results on hybrid functions (C10–C19, dimension = 30).

		DE	SSA	SCA	MFO	WOA	DBO
C10	Ave	2.3824 × 10^5^	2.5356 × 10^5^	3.8235 × 10^6^	7.4495 × 10^5^	3.0101 × 10^5^	1.9390 × 10^6^
	Std	7.5769 × 10^4^	2.5043 × 10^5^	3.1173 × 10^6^	9.0509 × 10^5^	4.3615 × 10^5^	1.8982 × 10^6^
	Rank	3	4	10	8	5	9
C11	Ave	4.1393 × 10^8^	5.3388 × 10^8^	3.1337 × 10^9^	1.9391 × 10^9^	1.0405 × 10^9^	4.3244 × 10^8^
	Std	1.2903 × 108	5.4696 × 10^8^	9.8563 × 10^8^	1.7738 × 10^9^	8.5502 × 10^8^	8.5984 × 10^8^
	Rank	3	5	11	9	7	4
C12	Ave	1.3180 × 10^8^	1.0432 × 10^9^	2.2508 × 10^9^	1.2072 × 10^9^	8.6726 × 10^8^	1.3450 × 10^9^
	Std	9.0952 × 10^7^	1.2250 × 10^9^	8.3328 × 10^8^	1.4706 × 10^9^	4.8556 × 10^8^	2.6294 × 10^9^
	Rank	3	6	10	7	5	9
C13	Ave	6.0983 × 10^5^	3.7275 × 10^6^	2.7138 × 10^6^	1.4532 × 10^6^	1.9689 × 10^6^	1.3957 × 10^6^
	Std	3.0735 × 10^5^	3.8287 × 10^6^	1.4229 × 10^6^	1.9387 × 10^6^	1.2815 × 10^6^	1.3469 × 10^6^
	Rank	3	11	9	6	7	5
C14	Ave	5.2572 × 10^7^	3.1892 × 10^8^	7.2280 × 10^8^	1.2978 × 10^8^	2.0984 × 10^8^	1.4844 × 10^8^
	Std	4.1440 × 10^7^	4.0858 × 10^8^	3.4740 × 10^8^	3.3414 × 10^8^	2.0304 × 10^8^	5.0534 × 10^8^
	Rank	3	9	10	5	7	6
C15	Ave	2.4606 × 10^4^	2.4629 × 10^7^	2.7235 × 10^7^	8.1481 × 10^7^	1.0801 × 10^7^	9.4404 × 10^7^
	Std	1.0443 × 104	8.9424 × 10^7^	3.0023 × 10^7^	1.3306 × 10^8^	1.2275 × 10^7^	1.6710 × 10^8^
	Rank	3	6	7	9	5	10
C16	Ave	1.2704 × 10^7^	2.9113 × 10^12^	5.8732 × 10^9^	1.3321 × 10^8^	4.6590 × 10^7^	2.9759 × 10^8^
	Std	2.7267 × 10^7^	1.0842 × 10^13^	1.0318 × 10^10^	7.1205 × 10^8^	1.1157 × 10^8^	1.4980 × 10^9^
	Rank	3	11	9	6	5	8
C17	Ave	2.5583 × 10^5^	1.2008 × 10^5^	8.7192 × 10^5^	8.7138 × 10^5^	4.0092 × 10^5^	2.7430 × 10^6^
	Std	7.7286 × 10^4^	4.9587 × 10^4^	6.1177 × 10^5^	2.1572 × 10^6^	4.0770 × 10^5^	5.9845 × 10^6^
	Rank	4	3	8	7	6	12
C18	Ave	2.6120 × 10^7^	1.1889 × 10^11^	1.0677 × 10^11^	6.0959 × 10^10^	2.1192 × 10^10^	3.3961 × 10^11^
	Std	1.7854 × 10^7^	5.5510 × 10^11^	2.9388 × 10^11^	5.2927 × 10^10^	2.3646 × 10^10^	6.6162 × 10^11^
	Rank	3	8	7	6	5	10
C19	Ave	4.4672 × 10^3^	1.1472 × 10^4^	5.8916 × 10^3^	4.4471 × 10^3^	5.2497 × 10^3^	4.9749 × 10^3^
	Std	5.6679 × 10^2^	2.8782 × 10^3^	1.0770 × 10^3^	2.9891 × 10^3^	9.9786 × 10^2^	1.3788 × 10^3^
	Rank	4	11	8	3	9	7
		**POA**	**SOA**	**BWO**	**GJO**	**NGO**	**MSINGO**
C10	Ave	1.2174 × 10^7^	4.5419 × 10^7^	6.5171 × 10^5^	4.7743 × 10^5^	3.5772 × 10^4^	**1.6519 × 10** ^4^
	Std	2.0078 × 10^7^	4.9249 × 10^7^	3.6771 × 10^5^	1.2237 × 10^6^	2.3458 × 10^4^	**8.7681 × 10** ^3^
	Rank	11	12	7	6	2	**1**
C11	Ave	1.4374 × 10^10^	8.3053 × 10^9^	6.8625 × 10^8^	1.0873 × 10^9^	1.9569 × 10^5^	**8.1669 × 10** ^4^
	Std	4.3551 × 10^9^	3.2900 × 10^9^	2.3423 × 10^8^	8.3723 × 10^8^	1.9607 × 10^5^	**5.8853 × 10** ^4^
	Rank	12	10	6	8	2	**1**
C12	Ave	1.6366 × 10^10^	1.0709 × 10^10^	2.4212 × 10^8^	1.2453 × 10^9^	2.5452 × 10^4^	**7.6979 × 10** ^3^
	Std	2.5142 × 10^9^	5.2024 × 10^9^	9.0134 × 10^7^	8.0927 × 10^8^	2.6804 × 10^4^	**5.5292 × 10** ^3^
	Rank	12	11	4	8	2	**1**
C13	Ave	1.0717 × 10^6^	2.0740 × 10^7^	2.4878 × 10^6^	3.6762 × 10^6^	2.5841 × 10^5^	**1.5022 × 10** ^5^
	Std	7.0693 × 10^5^	2.4172 × 10^7^	1.8788 × 10^6^	1.8478 × 10^6^	1.4319 × 10^5^	**4.4144 × 10** ^4^
	Rank	4	12	8	10	2	**1**
C14	Ave	7.1133 × 10^9^	5.8169 × 10^9^	5.6701 × 10^7^	2.8762 × 10^8^	2.6773 × 10^4^	**1.3331 × 10** ^4^
	Std	2.3403 × 10^9^	3.2930 × 10^9^	2.7721 × 10^7^	2.2670 × 10^8^	1.0410 × 10^4^	**7.8585 × 10** ^3^
	Rank	12	11	4	8	2	**1**
C15	Ave	1.9921 × 10^9^	1.7567 × 10^9^	1.7753 × 10^6^	3.3905 × 10^7^	1.9647 × 10^3^	**1.9586 × 10** ^3^
	Std	2.2691 × 10^9^	2.2460 × 10^9^	1.3368 × 10^6^	2.2802 × 10^7^	3.4647 × 10^2^	**3.6559 × 10** ^2^
	Rank	12	11	4	8	2	**1**
C16	Ave	3.0927 × 10^11^	1.3140 × 10^15^	3.7858 × 10^7^	1.7921 × 10^8^	7.3627 × 10^3^	**4.4896 × 10** ^3^
	Std	8.1938 × 10^11^	3.2889 × 10^15^	6.9027 × 10^7^	4.1231 × 10^8^	7.1224 × 10^3^	**2.6157 × 10** ^3^
	Rank	10	12	4	7	2	**1**
C17	Ave	3.8567 × 10^5^	1.2542 × 10^6^	1.9249 × 10^6^	1.1930 × 10^6^	9.2979 × 10^4^	**8.5337 × 10** ^4^
	Std	2.8337 × 10^5^	6.3729 × 10^5^	2.4965 × 10^6^	1.2711 × 10^6^	4.5633 × 10^4^	**3.0722 × 10** ^4^
	Rank	5	10	11	9	2	**1**
C18	Ave	3.2735 × 10^12^	6.7178 × 10^14^	2.1412 × 10^9^	1.6957 × 10^11^	**1.7265 × 10** ^4^	2.2163 × 10^4^
	Std	1.3036 × 10^13^	1.5232 × 10^15^	1.1168 × 10^9^	3.3946 × 10^11^	**1.7684 × 10** ^4^	1.7829 × 10^4^
	Rank	11	12	4	9	**1**	2
C19	Ave	1.3295 × 10^4^	1.0568 × 10^4^	4.4772 × 10^3^	4.9503 × 10^3^	2.7377 × 10^3^	**2.2469 × 10** ^3^
	Std	3.0948 × 10^3^	2.6512 × 10^3^	6.9120 × 10^2^	7.2511 × 10^2^	2.7274 × 10^2^	**1.6479 × 10** ^2^
	Rank	12	10	5	6	2	**1**

Bold is the best result of all algorithms.

**Table 12:** Comparison results on composition functions (C20–C29, dimension = 30).

		DE	SSA	SCA	MFO	WOA	DBO
C20	Ave	6.0889 × 10^3^	2.2039 × 10^4^	1.5167 × 10^4^	1.6832 × 10^4^	1.0976 × 10^4^	1.0256 × 10^4^
	Std	2.3964 × 10^3^	9.6740 × 10^3^	6.1198 × 10^3^	1.3998 × 10^4^	4.6101 × 10^3^	6.2115 × 10^3^
	Rank	3	10	8	9	7	5
C21	Ave	2.3752 × 10^3^	4.2211 × 10^3^	2.6210 × 10^3^	2.5464 × 10^3^	2.4818 × 10^3^	2.6688 × 10^3^
	Std	1.4146 × 10^1^	6.4724 × 10^2^	8.0512 × 10^1^	1.0171 × 10^2^	5.4961 × 10^1^	1.6943 × 10^2^
	Rank	3	10	8	7	6	9
C22	Ave	1.1479 × 10^4^	3.3571 × 10^4^	2.6421 × 10^4^	2.1697 × 10^4^	1.8355 × 10^4^	1.8080 × 10^4^
	Std	8.7594 × 10^2^	8.7025 × 10^3^	3.8245 × 10^3^	8.7977 × 10^3^	3.0176 × 10^3^	5.0424 × 10^3^
	Rank	3	10	9	8	7	6
C23	Ave	8.0055 × 10^3^	2.1791 × 10^4^	1.7674 × 10^4^	1.2769 × 10^4^	1.3186 × 10^4^	1.0497 × 10^4^
	Std	5.3995 × 10^2^	4.8587 × 10^3^	2.6056 × 10^3^	3.4069 × 10^3^	2.7605 × 10^3^	2.5129 × 10^3^
	Rank	3	10	9	7	8	4
C24	Ave	3.1773 × 10^3^	3.9174 × 10^3^	3.8390 × 10^3^	3.9923 × 10^3^	3.4682 × 10^3^	3.2048 × 10^3^
	Std	1.1303 × 10^2^	3.3045 × 10^2^	2.4359 × 10^2^	9.2974 × 10^2^	2.3489 × 10^2^	3.8471 × 10^2^
	Rank	4	9	8	10	7	5
C25	Ave	3.3657 × 10^3^	6.1522 × 10^3^	4.4970 × 10^3^	3.4268 × 10^3^	3.6861 × 10^3^	3.8545 × 10^3^
	Std	1.4826 × 10^1^	1.3863 × 10^3^	4.1089 × 10^2^	4.1888 × 10^1^	1.2616 × 10^2^	5.3122 × 10^2^
	Rank	3	10	9	5	6	8
C26	Ave	3.1445 × 10^3^	4.1549 × 10^3^	3.4964 × 10^3^	3.1968 × 10^3^	3.3498 × 10^3^	3.2549 × 10^3^
	Std	1.2613 × 10^1^	5.5968 × 10^2^	7.2717 × 10^1^	4.2376 × 10^1^	5.9534 × 10^1^	9.5293 × 10^1^
	Rank	3	10	9	4	8	6
C27	Ave	3.3147 × 10^3^	4.1378 × 10^3^	3.9593 × 10^3^	3.8085 × 10^3^	3.5068 × 10^3^	4.1153 × 10^3^
	Std	3.6930 × 10^1^	5.1463 × 10^2^	2.1656 × 10^2^	1.6789 × 10^2^	1.4454 × 10^2^	4.6185 × 10^2^
	Rank	3	10	8	7	5	9
C28	Ave	3.9188 × 10^8^	4.1002 × 10^9^	8.4025 × 10^9^	1.8019 × 10^10^	1.0726 × 10^9^	8.3308 × 10^8^
	Std	4.2525 × 10^8^	6.3386 × 10^9^	9.6860 × 10^9^	9.5223 × 10^10^	7.2428 × 10^8^	1.4082 × 10^9^
	Rank	3	8	9	10	6	5
C29	Ave	3.1419 × 10^8^	1.2640 × 10^10^	6.0558 × 10^9^	8.6999 × 10^9^	2.9297 × 10^9^	8.0939 × 10^10^
	Std	1.6203 × 10^8^	4.0154 × 10^10^	3.2377 × 10^9^	1.1304 × 10^10^	1.9782 × 10^9^	2.8023 × 10^11^
	Rank	3	9	7	8	5	10
		**POA**	**SOA**	**BWO**	**GJO**	**NGO**	**MSINGO**
C20	Ave	3.0832 × 10^4^	5.1253 × 10^4^	6.4274 × 10^3^	1.0731 × 10^4^	2.6466 × 10^3^	**2.3231 × 10** ^3^
	Std	1.2701 × 10^4^	8.9562 × 10^3^	2.5167 × 10^3^	3.1797 × 10^3^	4.8847 × 10^2^	**1.5802 × 10** ^2^
	Rank	11	12	4	6	2	**1**
C21	Ave	4.9915 × 10^3^	5.1938 × 10^3^	2.4040 × 10^3^	2.4158 × 10^3^	2.3416 × 10^3^	**2.2752 × 10** ^3^
	Std	1.3506 × 10^3^	9.4591 × 10^2^	2.6864 × 10^1^	2.6154 × 10^1^	2.7963 × 10^1^	**6.6923**
	Rank	11	12	4	5	2	**1**
C22	Ave	5.7229 × 10^4^	6.3209 × 10^4^	1.3620 × 10^4^	1.7769 × 10^4^	3.0030 × 10^3^	**2.4422 × 10** ^3^
	Std	9.2948 × 10^3^	6.3493 × 10^3^	1.5893 × 10^3^	1.9906 × 10^3^	5.7231 × 10^2^	**1.1063 × 10** ^2^
	Rank	11	12	4	5	2	**1**
C23	Ave	3.2591 × 10^4^	3.3602 × 10^4^	1.0588 × 10^4^	1.2191 × 10^4^	2.8273 × 10^3^	**2.5146 × 10** ^3^
	Std	3.7133 × 10^3^	2.8012 × 10^3^	1.7990 × 10^3^	1.2763 × 10^3^	4.6000 × 10^2^	**6.2048 × 10** ^1^
	Rank	11	12	5	6	2	**1**
C24	Ave	7.3834 × 10^3^	7.5119 × 10^3^	3.1730 × 10^3^	3.3755 × 10^3^	2.8767 × 10^3^	**2.8249 × 10** ^3^
	Std	1.0436 × 10^3^	9.2234 × 10^2^	7.6243 × 10^1^	8.6234 × 10^1^	2.2767 × 10^1^	**4.6406**
	Rank	11	12	3	6	2	**1**
C25	Ave	9.1076 × 10^3^	1.2374 × 10^4^	3.4414 × 10^3^	3.7395 × 10^3^	3.3543 × 10^3^	**3.3352 × 10** ^3^
	Std	2.6356 × 10^3^	6.2058 × 10^3^	5.6777 × 10^1^	1.1559 × 10^2^	1.8431 × 10^1^	**1.3340 × 10** ^1^
	Rank	11	12	4	7	2	**1**
C26	Ave	3.5546 × 10^3^	4.3146 × 10^3^	3.1969 × 10^3^	3.3483 × 10^3^	3.1376 × 10^3^	**3.1284 × 10** ^3^
	Std	1.6801 × 10^2^	3.8833 × 10^2^	1.9053 × 10^1^	4.3705 × 10^1^	2.1172 × 10^1^	**1.9031 × 10** ^1^
	Rank	11	12	5	7	2	**1**
C27	Ave	7.8579 × 10^3^	6.3553 × 10^3^	3.3934 × 10^3^	3.5350 × 10^3^	2.9989 × 10^3^	**2.7338 × 10** ^3^
	Std	1.4815 × 10^3^	1.5897 × 10^3^	6.9237 × 10^1^	1.2377 × 10^2^	1.3136 × 10^2^	**4.6613 × 10** ^1^
	Rank	12	11	4	6	2	**1**
C28	Ave	4.0327 × 10^11^	7.0928 × 10^12^	4.2329 × 10^8^	2.8629 × 10^9^	3.8901 × 10^4^	**1.7619 × 10** ^4^
	Std	7.4454 × 10^11^	1.6879 × 10^13^	4.7836 × 10^8^	1.5993 × 10^9^	3.8092 × 10^4^	**1.5936 × 10** ^4^
	Rank	11	12	4	7	2	**1**
C29	Ave	1.6043 × 10^11^	1.5796 × 10^12^	4.9480 × 10^8^	3.4954 × 10^9^	7.7663 × 10^4^	**4.2697 × 10** ^4^
	Std	2.5786 × 10^11^	3.7520 × 10^12^	3.0719 × 10^8^	1.0517 × 10^9^	7.1995 × 10^4^	**6.2395 × 10** ^4^
	Rank	11	12	4	6	2	**1**

Bold is the best result of all algorithms.

**Table 13:** The *p*-values from Wilcoxon signed-rank test on hybrid and composition functions (C10–C29, dimension = 30).

Ours vs.	DE	SSA	SCA	MFO	WOA	DBO	POA	SOA	BWO	GJO	NGO
C10	2.87 × 10^−11^	2.87 × 10^−11^	2.87 × 10^−11^	3.51 × 10^−11^	2.87 × 10^−11^	2.87 × 10^−11^	2.87 × 10^−11^	2.87 × 10^−11^	2.87 × 10^−11^	2.87 × 10^−11^	4.58 × 10^−4^
C11	2.87 × 10^−11^	2.87 × 10^−11^	2.87 × 10^−11^	2.87 × 10^−11^	2.87 × 10^−11^	2.87 × 10^−11^	2.87 × 10^−11^	2.87 × 10^−11^	2.87 × 10^−11^	2.87 × 10^−11^	7.13 × 10^−4^
C12	2.87 × 10^−11^	2.87 × 10^−11^	2.87 × 10^−11^	2.87 × 10^−11^	2.87 × 10^−11^	2.87 × 10^−11^	2.87 × 10^−11^	2.87 × 10^−11^	2.87 × 10^−11^	2.87 × 10^−11^	2.98 × 10^−6^
C13	3.51 × 10^−11^	3.51 × 10^−11^	2.87 × 10^−11^	2.87 × 10^−11^	2.87 × 10^−11^	2.87 × 10^−11^	2.87 × 10^−11^	2.87 × 10^−11^	2.87 × 10^−11^	2.87 × 10^−11^	1.54 × 10^−4^
C14	2.87 × 10^−11^	2.87 × 10^−11^	2.87 × 10^−11^	2.87 × 10^−11^	2.87 × 10^−11^	2.87 × 10^−11^	2.87 × 10^−11^	2.87 × 10^−11^	2.87 × 10^−11^	2.87 × 10^−11^	2.58 × 10^−6^
C15	2.87 × 10^−11^	2.87 × 10^−11^	2.87 × 10^−11^	2.87 × 10^−11^	2.87 × 10^−11^	2.87 × 10^−11^	2.87 × 10^−11^	2.87 × 10^−11^	2.87 × 10^−11^	2.87 × 10^−11^	8.59 × 10^−1^
C16	2.87 × 10^−11^	2.87 × 10^−11^	2.87 × 10^−11^	2.87 × 10^−11^	2.87 × 10^−11^	2.87 × 10^−11^	2.87 × 10^−11^	2.87 × 10^−11^	2.87 × 10^−11^	2.87 × 10^−11^	4.51 × 10^−1^
C17	3.51 × 10^−11^	4.97 × 10^−11^	2.87 × 10^−11^	2.13 × 10^−9^	3.13 × 10^−7^	7.03 × 10^−11^	3.88 × 10^−11^	5.23 × 10^−11^	3.88 × 10^−11^	2.05 × 10^−11^	6.68 × 10^−1^
C18	2.87 × 10^−11^	2.87 × 10^−11^	2.87 × 10^−11^	2.87 × 10^−11^	2.87 × 10^−11^	2.87 × 10^−11^	2.87 × 10^−11^	2.87 × 10^−11^	2.87 × 10^−11^	2.87 × 10^−11^	3.08 × 10^−1^
C19	2.87 × 10^−11^	2.87 × 10^−11^	2.87 × 10^−11^	7.44 × 10^−9^	2.87 × 10^−11^	2.87 × 10^−11^	2.87 × 10^−11^	2.87 × 10^−11^	2.87 × 10^−11^	2.87 × 10^−11^	1.94 × 10^−9^
C20	2.87 × 10^−11^	2.87 × 10^−11^	2.87 × 10^−11^	2.87 × 10^−11^	2.87 × 10^−11^	2.87 × 10^−11^	2.87 × 10^−11^	2.87 × 10^−11^	2.87 × 10^−11^	2.87 × 10^−11^	2.19 × 10-^2^
C21	2.87 × 10^−11^	2.87 × 10^−11^	2.87 × 10^−11^	2.87 × 10^−11^	2.87 × 10^−11^	2.87 × 10^−11^	2.87 × 10^−11^	2.87 × 10^−11^	2.87 × 10^−11^	2.87 × 10^−11^	3.18 × 10^−11^
C22	2.87 × 10^−11^	2.87 × 10^−11^	2.87 × 10^−11^	2.87 × 10^−11^	2.87 × 10^−11^	2.87 × 10^−11^	2.87 × 10^−11^	2.87 × 10^−11^	2.87 × 10^−11^	2.87 × 10^−11^	3.06 × 10^−9^
C23	2.87 × 10^−11^	2.87 × 10^−11^	2.87 × 10^−11^	2.87 × 10^−11^	2.87 × 10^−11^	2.87 × 10^−11^	2.87 × 10^−11^	2.87 × 10^−11^	2.87 × 10^−11^	2.87 × 10^−11^	2.74 × 10^−10^
C24	2.87 × 10^−11^	2.87 × 10^−11^	2.87 × 10^−11^	2.87 × 10^−11^	2.87 × 10^−11^	2.87 × 10^−11^	2.87 × 10^−11^	2.87 × 10^−11^	2.87 × 10^−11^	2.87 × 10^−11^	2.87 × 10^−11^
C25	8.86 × 10^−11^	2.87 × 10^−11^	2.87 × 10^−11^	2.87 × 10^−11^	2.87 × 10^−11^	2.87 × 10^−11^	2.87 × 10^−11^	2.87 × 10^−11^	2.87 × 10^−11^	2.87 × 10^−11^	1.67 × 10^−6^
C26	1.07 × 10^−11^	2.87 × 10^−11^	2.87 × 10^−11^	2.87 × 10^−11^	2.87 × 10^−11^	2.87 × 10^−11^	2.87 × 10^−11^	2.87 × 10^−11^	6.37 × 10^−11^	2.87 × 10^−11^	7.13 × 10^−2^
C27	2.87 × 10^−11^	2.87 × 10^−11^	2.87 × 10^−11^	2.87 × 10^−11^	2.87 × 10^−11^	2.87 × 10^−11^	2.87 × 10^−11^	2.87 × 10^−11^	2.87 × 10^−11^	2.87 × 10^−11^	1.54 × 10^−10^
C28	2.87 × 10^−11^	2.87 × 10^−11^	2.87 × 10^−11^	2.87 × 10^−11^	2.87 × 10^−11^	2.87 × 10^−11^	2.87 × 10^−11^	2.87 × 10^−11^	2.87 × 10^−11^	2.87 × 10^−11^	3.26 × 10^−5^
C29	2.87 × 10^−11^	2.87 × 10^−11^	2.87 × 10^−11^	2.87 × 10^−11^	2.87 × 10^−11^	2.87 × 10^−11^	2.87 × 10^−11^	2.87 × 10^−11^	2.87 × 10^−11^	2.87 × 10^−11^	5.79 × 10^−5^

**Table 14:** Comparison results of 12 algorithms on CEC2017 (dimension = 100).

		DE	SSA	SCA	MFO	WOA	DBO
C1	Ave	1.8714 × 10^11^	2.2451 × 10^11^	2.1794 × 10^11^	2.0379 × 10^11^	1.5912 × 10^11^	8.9114 × 10^10^
	Std	3.3258 × 10^10^	1.9344 × 10^10^	1.3744 × 10^10^	5.5019 × 10^10^	1.7294 × 10^10^	1.9767 × 10^10^
	Rank	7	10	9	8	6	3
C2	Ave	5.8117 × 10^5^	3.4224 × 10^5^	2.9727 × 10^5^	4.6114 × 10^5^	2.3816 × 10^5^	3.6726 × 10^5^
	Std	5.0427 × 10^4^	1.5552 × 10^4^	2.4056 × 10^4^	1.0256 × 10^5^	1.8431 × 10^4^	9.1226 × 10^4^
	Rank	12	7	5	11	3	8
C3	Ave	3.1431 × 10^4^	8.7861 × 10^4^	5.8632 × 10^4^	5.0840 × 10^4^	2.7590 × 10^4^	2.2050 × 10^4^
	Std	6.0826 × 10^3^	1.6582 × 10^4^	9.8769 × 10^3^	2.8771 × 10^4^	6.0483 × 10^3^	1.2966 × 10^4^
	Rank	7	10	9	8	5	4
C4	Ave	1.7912 × 10^5^	2.8037 × 10^5^	2.3375 × 10^5^	2.3947 × 10^5^	1.6200 × 10^5^	9.1679 × 10^4^
	Std	2.0952 × 10^4^	2.3223 × 10^4^	1.6550 × 10^4^	6.1382 × 10^4^	1.8946 × 10^4^	2.0503 × 10^4^
	Rank	7	10	8	9	6	3
C5	Ave	5.0013 × 10^2^	5.0004 × 10^2^	5.0008 × 10^2^	5.0002 × 10^2^	5.0003 × 10^2^	5.0007 × 10^2^
	Std	1.6821 × 10^−2^	1.1768 × 10^−2^	1.1816 × 10^−2^	7.0367 × 10^−3^	7.7224 × 10^−3^	2.1163 × 10^−2^
	Rank	12	7	11	3	5	10
C6	Ave	1.8110 × 10^5^	5.2966 × 10^4^	1.2772 × 10^5^	4.9471 × 10^4^	7.0580 × 10^4^	1.0061 × 10^5^
	Std	2.1493 × 10^4^	1.1622 × 10^4^	1.5073 × 10^4^	1.5183 × 10^4^	1.1143 × 10^4^	1.5069 × 10^4^
	Rank	12	3	10	2	6	8
C7	Ave	7.1334 × 10^2^	7.0367 × 10^2^	7.0846 × 10^2^	7.0264 × 10^2^	7.0420 × 10^2^	7.0682 × 10^2^
	Std	1.6047	1.1108	1.2550	9.0368 × 10^−1^	8.9731 × 10^−1^	1.5332
	Rank	12	4	10	3	6	8
C8	Ave	1.0631 × 10^3^	1.0415 × 10^3^	1.0859 × 10^3^	1.0263 × 10^3^	9.8904 × 10^2^	1.0028 × 10^3^
	Std	3.5307 × 10^1^	3.3135 × 10^1^	3.9201 × 10^1^	3.6753 × 10^1^	1.8707 × 10^1^	3.0361 × 10^1^
	Rank	9	8	11	7	5	6
C9	Ave	3.3469 × 10^4^	2.5666 × 10^4^	3.3485 × 10^4^	2.3548 × 10^4^	2.9961 × 10^4^	3.1757 × 10^4^
	Std	6.3076 × 10^2^	1.3784 × 10^3^	6.2937 × 10^2^	1.3766 × 10^3^	1.3469 × 10^3^	1.2608 × 10^3^
	Rank	11	3	12	2	6	7
C10	Ave	1.0950 × 10^8^	5.2412 × 10^9^	9.6620 × 10^8^	1.7408 × 10^9^	3.9623 × 10^8^	1.0114 × 10^9^
	Std	4.6414 × 10^7^	2.6803 × 10^9^	4.1832 × 10^8^	2.2862 × 10^9^	3.6290 × 10^8^	1.7108 × 10^9^
	Rank	3	10	7	9	6	8
C11	Ave	3.9643 × 10^10^	1.6110 × 10^11^	9.8334 × 10^10^	6.6192 × 10^10^	5.7521 × 10^10^	4.5170 × 10^10^
	Std	6.0463 × 10^9^	1.6734 × 10^10^	9.9422 × 10^9^	2.3068 × 10^10^	1.5850 × 10^10^	1.4588 × 10^10^
	Rank	3	10	9	8	7	4
C12	Ave	6.3041 × 10^10^	2.5220 × 10^11^	1.6476 × 10^11^	1.2474 × 10^11^	8.8210 × 10^10^	5.2968 × 10^10^
	Std	1.3926 × 10^10^	3.9817 × 10^10^	2.5106 × 10^10^	7.7464 × 10^10^	2.5571 × 10^10^	2.5852 × 10^10^
	Rank	4	10	9	8	7	3
C13	Ave	9.0632 × 10^7^	1.5284 × 10^8^	1.0641 × 10^8^	3.6619 × 10^7^	3.2953 × 10^7^	3.9380 × 10^7^
	Std	1.8954 × 10^7^	9.4472 × 10^7^	3.5987 × 10^7^	2.9756 × 10^7^	1.3111 × 10^7^	2.9826 × 10^7^
	Rank	8	10	9	5	3	6
C14	Ave	1.7157 × 10^10^	4.1575 × 10^10^	6.4363 × 10^9^	2.1118 × 10^10^	1.3052 × 10^10^	5.3754 × 10^9^
	Std	4.6542 × 10^9^	6.4363 × 10^9^	5.5286 × 10^9^	1.5106 × 10^10^	3.5989 × 10^9^	7.7236 × 10^9^
	Rank	7	10	9	8	6	3
C15	Ave	2.7372 × 10^8^	9.3648 × 10^9^	3.5003 × 10^9^	1.3216 × 10^9^	1.6270 × 10^9^	5.6257 × 10^8^
	Std	1.6406 × 10^8^	3.1402 × 10^9^	1.2131 × 10^9^	1.5564 × 10^9^	1.1732 × 10^9^	1.1270 × 10^9^
	Rank	3	10	9	7	8	4
C16	Ave	1.6080 × 10^13^	2.1785 × 10^15^	1.5784 × 10^14^	3.4409 × 10^12^	1.7698 × 10^13^	8.0268 × 10^12^
	Std	1.4585 × 1013	2.1762 × 1015	1.4949 × 1014	6.3994 × 1012	2.7751 × 10^13^	2.4554 × 10^13^
	Rank	7	10	9	4	8	6
C17	Ave	4.7197 × 10^7^	3.5049 × 10^8^	7.8418 × 10^7^	2.3595 × 10^7^	2.5945 × 10^7^	3.7904 × 10^7^
	Std	1.4321 × 10^7^	4.1373 × 10^8^	3.1270 × 10^7^	1.4758 × 10^7^	1.5739 × 10^7^	3.1630 × 10^7^
	Rank	7	11	9	3	4	6
C18	Ave	1.2987 × 10^11^	1.5637 × 10^15^	5.4805 × 10^13^	3.4544 × 10^13^	2.3872 × 10^13^	4.9242 × 10^13^
	Std	4.8343 × 10^10^	1.1323 × 10^15^	5.6232 × 10^13^	6.7410 × 10^13^	4.6587 × 10^13^	1.7240 × 10^14^
	Rank	3	10	9	7	6	8
C19	Ave	1.4185 × 10^4^	4.0968 × 10^4^	2.0195 × 10^4^	1.8634 × 10^4^	1.8613 × 10^4^	1.5225 × 10^4^
	Std	1.2408 × 10^3^	4.6721 × 10^3^	2.9459 × 10^3^	9.0316 × 10^3^	3.1425 × 10^3^	3.2202 × 10^3^
	Rank	4	12	9	8	7	6
C20	Ave	1.8601 × 10^4^	2.3097 × 10^4^	2.1652 × 10^5^	1.9596 × 10^5^	1.5790 × 10^5^	1.0071 × 10^5^
	Std	2.9660 × 10^4^	1.3891 × 10^4^	1.4003 × 10^4^	4.3676 × 10^4^	1.9050 × 10^4^	2.1569 × 10^4^
	Rank	7	10	9	8	6	3
C21	Ave	4.2211 × 10^3^	2.4411 × 10^4^	1.4136 × 10^4^	1.5069 × 10^4^	7.1533 × 10^3^	1.2942 × 10^4^
	Std	4.8456 × 10^3^	2.6404 × 10^3^	2.4611 × 10^3^	9.0638 × 10^3^	1.6881 × 10^3^	1.0667 × 10^4^
	Rank	2	10	8	9	6	7
C22	Ave	6.2058 × 10^4^	1.2499 × 10^5^	1.2821 × 10^5^	8.1417 × 10^4^	1.1296 × 10^5^	7.5481 × 10^4^
	Std	6.4505 × 10^3^	2.8138 × 10^3^	5.0529 × 10^3^	1.9281 × 10^4^	2.7483 × 10^3^	1.5720 × 10^4^
	Rank	2	9	10	5	8	3
C23	Ave	7.9316 × 10^4^	1.6838 × 10^5^	1.6689 × 10^5^	1.1732 × 10^5^	1.4457 × 10^5^	9.0111 × 10^4^
	Std	9.7544 × 10^3^	4.7708 × 10^3^	9.1002 × 10^3^	4.5696 × 10^4^	1.0299 × 10^4^	2.1932 × 10^4^
	Rank	2	10	9	6	8	3
C24	Ave	1.9742 × 10^4^	2.8569 × 10^4^	2.5740 × 10^4^	2.3018 × 10^4^	1.3254 × 10^4^	9.1553 × 10^3^
	Std	3.6475 × 10^3^	4.3503 × 10^3^	3.5689 × 10^3^	1.0784 × 10^4^	1.9038 × 10^3^	1.9815 × 10^3^
	Rank	7	10	9	8	5	3
C25	Ave	7.8815 × 10^3^	1.9336 × 10^5^	8.1783 × 10^4^	9.6644 × 10^3^	3.6022 × 10^4^	1.7992 × 10^4^
	Std	5.4080 × 10^2^	7.4500 × 10^4^	1.2994 × 10^4^	1.7413 × 10^3^	8.9004 × 10^3^	6.0362 × 10^3^
	Rank	2	10	9	4	7	6
C26	Ave	4.1844 × 10^3^	1.0527 × 10^4^	7.4291 × 10^3^	4.3089 × 10^3^	5.7508 × 10^3^	4.7032 × 10^3^
	Std	2.1289 × 10^2^	1.3710 × 10^3^	4.7861 × 10^2^	3.2853 × 10^2^	3.4251 × 10^2^	5.9115 × 10^2^
	Rank	3	11	10	4	7	5
C27	Ave	7.8695 × 10^3^	2.1520 × 10^4^	1.5464 × 10^4^	9.5747 × 10^3^	8.9684 × 10^3^	8.2506 × 10^3^
	Std	6.5597 × 10^2^	2.9496 × 10^3^	1.8744 × 10^3^	1.2426 × 10^3^	1.3524 × 10^3^	2.6792 × 10^3^
	Rank	3	10	9	8	6	4
C28	Ave	4.2680 × 10^12^	1.8533 × 10^15^	1.2952 × 10^14^	1.2616 × 10^15^	9.7234 × 10^13^	4.9022 × 10^13^
	Std	4.7020 × 10^12^	1.9674 × 10^15^	1.1273 × 10^14^	3.3475 × 10^15^	1.2558 × 10^14^	1.5075 × 10^14^
	Rank	3	10	8	9	7	6
C29	Ave	4.5676 × 10^12^	9.1778 × 10^14^	8.3903 × 10^13^	9.0060 × 10^14^	6.5671 × 10^13^	4.3255 × 10^13^
	Std	3.7867 × 10^12^	8.7941 × 10^14^	8.0805 × 10^13^	1.6666 × 10^15^	6.8414 × 10^13^	1.3850 × 10^14^
	Rank	4	10	8	9	7	6
		**POA**	**SOA**	**BWO**	**GJO**	**NGO**	**MSINGO**
C1	Ave	2.7197 × 10^11^	2.6571 × 10^11^	1.4584 × 10^11^	1.4130 × 10^11^	5.4890 × 10^10^	**8.4928 × 10** ^8^
	Std	6.9345 × 10^9^	1.1789 × 10^10^	1.3582 × 10^10^	1.1035 × 10^10^	9.7426 × 10^9^	**1.6473 × 10** ^8^
	Rank	12	11	5	4	2	**1**
C2	Ave	4.4141 × 10^5^	4.4752 × 10^5^	3.3394 × 10^5^	2.4770 × 10^5^	1.0009 × 10^5^	**5.5465 × 10** ^4^
	Std	9.2720 × 10^4^	9.7072 × 10^4^	2.5787 × 10^4^	2.0284 × 10^4^	1.0894 × 10^4^	**8.4001 × 10** ^3^
	Rank	9	10	6	4	2	**1**
C3	Ave	1.3761 × 10^5^	1.2197 × 10^5^	2.8413 × 10^4^	2.0899 × 10^4^	5.5971 × 10^3^	**1.0925 × 10** ^3^
	Std	1.3293 × 10^4^	1.7944 × 10^4^	5.0326 × 10^3^	3.4556 × 10^3^	1.3988 × 10^3^	**1.0775 × 10** ^2^
	Rank	12	11	6	3	2	**1**
C4	Ave	3.4659 × 10^5^	3.3982 × 10^5^	1.4924 × 10^5^	1.3076 × 10^5^	5.4008 × 10^4^	**2.5471 × 10** ^3^
	Std	1.2819 × 10^4^	1.7820 × 10^4^	1.4383 × 10^4^	1.4710 × 10^4^	8.0657 × 10^3^	**2.2091 × 10** ^2^
	Rank	12	11	5	4	2	**1**
C5	Ave	5.0007 × 10^2^	5.0007 × 10^2^	5.0004 × 10^2^	5.0003 × 10^2^	5.0001 × 10^2^	**5.0001 × 10** ^2^
	Std	1.6995 × 10^−2^	1.0127 × 10^−2^	7.9461 × 10^−3^	5.4425 × 10^−3^	5.8196 × 10^−3^	**3.2887 × 10** ^−3^
	Rank	9	8	6	4	2	**1**
C6	Ave	1.3280 × 10^5^	1.0459 × 10^5^	9.5042 × 10^4^	5.9436 × 10^4^	**2.1376 × 10** ^4^	6.0034 × 10^4^
	Std	1.0984 × 10^4^	1.1421 × 10^4^	8.8743 × 10^3^	1.1377 × 10^4^	**3.1109 × 10** ^3^	1.0923 × 10^4^
	Rank	11	9	7	4	**1**	5
C7	Ave	7.0894 × 10^2^	7.0714 × 10^2^	7.0615 × 10^2^	7.0381 × 10^2^	**7.0154 × 10** ^2^	7.0214 × 10^2^
	Std	1.2956	7.6338 × 10^−1^	8.9235 × 10^−1^	8.1803 × 10^−1^	**2.9539 × 10** ^−1^	3.1753 × 10^−1^
	Rank	11	9	7	5	**1**	2
C8	Ave	1.0757 × 10^3^	1.1257 × 10^3^	9.8844 × 10^2^	9.5851 × 10^2^	8.8063 × 10^2^	**8.3947 × 10** ^2^
	Std	2.6452 × 10^1^	3.1883 × 10^1^	2.9761 × 10^1^	1.4760 × 10^1^	9.2242	**8.9618**
	Rank	10	12	4	3	2	**1**
C9	Ave	3.2439 × 10^4^	3.2912 × 10^4^	3.2839 × 10^4^	2.9635 × 10^4^	**2.3546 × 10** ^4^	2.6675 × 10^4^
	Std	4.4694 × 10^2^	9.2632 × 10^2^	8.8386 × 10^2^	1.6070 × 10^3^	**8.4292 × 10** ^2^	9.3004 × 10^2^
	Rank	8	10	9	5	**1**	4
C10	Ave	1.3643 × 10^10^	1.2385 × 10^10^	1.1090 × 10^8^	3.8427 × 10^8^	3.2330 × 10^5^	**1.4126 × 10** ^5^
	Std	4.1704 × 10^9^	4.2609 × 10^9^	6.2048 × 10^7^	1.9565 × 10^8^	6.8403 × 10^4^	**4.7198 × 10** ^4^
	Rank	12	11	4	5	2	**1**
C11	Ave	2.1603 × 10^11^	2.0048 × 10^11^	4.9144 × 10^10^	5.2750 × 10^10^	3.5261 × 10^9^	**9.0874 × 10** ^7^
	Std	1.8518 × 10^10^	2.2306 × 10^10^	7.7498 × 10^9^	9.9203 × 10^9^	1.3724 × 10^9^	**2.6935 × 10** ^7^
	Rank	12	11	5	6	2	**1**
C12	Ave	3.9166 × 10^11^	3.6647 × 10^11^	7.4745 × 10^10^	6.9642 × 10^10^	5.2550 × 10^9^	**2.2443 × 10** ^7^
	Std	3.8587 × 10^10^	4.1229 × 10^10^	1.4011 × 10^10^	1.7967 × 10^10^	3.3866 × 10^9^	**1.2996 × 10** ^7^
	Rank	12	11	6	5	2	**1**
C13	Ave	1.8003 × 10^8^	5.5452 × 10^8^	4.6331 × 10^7^	3.3962 × 10^7^	3.1420 × 10^6^	**2.2309 × 10** ^6^
	Std	7.2055 × 10^7^	2.7697 × 10^8^	1.8832 × 10^7^	1.8692 × 10^7^	1.3169 × 10^6^	**1.2777 × 10** ^6^
	Rank	11	12	7	4	2	**1**
C14	Ave	6.3373 × 10^10^	5.6712 × 10^10^	9.3942 × 10^9^	9.8012 × 10^9^	1.5295 × 10^8^	**5.9354 × 10** ^5^
	Std	4.9593 × 10^9^	7.4869 × 10^9^	2.4456 × 10^9^	2.3664 × 10^9^	1.0588 × 10^8^	**8.2288 × 10** ^5^
	Rank	11	12	4	5	2	**1**
C15	Ave	1.9384 × 10^10^	1.4827 × 10^10^	5.9723 × 10^8^	6.6147 × 10^8^	2.0594 × 10^5^	**1.0052 × 10** ^4^
	Std	4.0925 × 10^9^	4.1885 × 10^9^	2.9038 × 10^8^	5.6126 × 10^8^	2.6539 × 10^5^	**4.0463 × 10** ^3^
	Rank	12	11	5	6	2	**1**
C16	Ave	1.0971 × 10^16^	1.2848 × 10^16^	4.4153 × 10^12^	2.4385 × 10^12^	3.4450 × 10^5^	**1.8396 × 10** ^4^
	Std	6.4344 × 10^15^	8.5564 × 10^15^	5.6616 × 10^12^	3.5274 × 10^12^	1.0012 × 10^6^	**1.2462 × 10** ^4^
	Rank	11	12	5	3	2	**1**
C17	Ave	8.7034 × 10^7^	9.1846 × 10^8^	5.4226 × 10^7^	3.1966 × 10^7^	2.8924 × 10^6^	**1.7515 × 10** ^6^
	Std	4.4478 × 10^7^	8.0554 × 10^8^	2.5658 × 10^7^	2.5626 × 10^7^	1.6674 × 10^6^	**8.3470 × 10** ^5^
	Rank	10	12	8	5	2	**1**
C18	Ave	5.3588 × 1015	5.4466 × 1015	9.2231 × 1011	1.8409 × 1012	1.6219 × 10^9^	**8.0681 × 10** ^4^
	Std	2.3777 × 10^15^	3.3979 × 10^15^	1.0244 × 10^12^	3.1529 × 10^12^	1.6709 × 10^9^	**3.9072 × 10** ^4^
	Rank	11	12	4	5	2	**1**
C19	Ave	3.9360 × 10^4^	3.5902 × 10^4^	1.4653 × 10^4^	1.2227 × 10^4^	1.0594 × 10^4^	**3.6227 × 10** ^3^
	Std	2.7796 × 10^3^	4.5031 × 10^3^	2.4324 × 10^3^	2.0835 × 10^3^	2.4325 × 10^3^	**6.7418 × 10** ^2^
	Rank	11	12	5	3	2	**1**
C20	Ave	2.6774 × 10^5^	2.5812 × 10^5^	1.5194 × 10^5^	1.3359 × 10^5^	6.6361 × 10^4^	**4.3931 × 10** ^3^
	Std	6.9321 × 10^3^	1.0314 × 10^4^	1.1221 × 10^4^	1.0917 × 10^4^	1.5980 × 10^4^	**2.6209 × 10** ^2^
	Rank	12	11	5	4	2	**1**
C21	Ave	3.0604 × 10^4^	2.8400 × 10^4^	4.9006 × 10^3^	5.3387 × 10^3^	4.2665 × 10^3^	**2.4277 × 10** ^3^
	Std	1.8045 × 10^3^	1.4014 × 10^3^	4.8344 × 10^2^	6.1843 × 10^2^	1.2618 × 10^3^	**1.9240 × 10** ^1^
	Rank	12	11	4	5	3	**1**
C22	Ave	1.2974 × 10^5^	1.2954 × 10^5^	1.0658 × 10^5^	7.8477 × 10^4^	8.1696 × 10^4^	**5.9174 × 10** ^3^
	Std	1.1120 × 10^3^	2.1654 × 10^3^	2.2259 × 10^3^	7.6809 × 10^3^	8.4532 × 10^3^	**1.0142 × 10** ^3^
	Rank	12	11	7	4	6	**1**
C23	Ave	1.8700 × 10^5^	1.8323 × 10^5^	1.3544 × 10^5^	1.0518 × 10^5^	9.2968 × 10^4^	**1.0313 × 10** ^4^
	Std	2.9026 × 10^3^	6.8315 × 10^3^	4.7580 × 10^3^	1.1031 × 10^4^	1.1393 × 10^4^	**2.3948 × 10** ^3^
	Rank	12	11	7	5	4	**1**
C24	Ave	4.7859 × 10^4^	4.1915 × 10^4^	1.4354 × 10^4^	1.2213 × 10^4^	6.6933 × 10^3^	**3.9888 × 10** ^3^
	Std	4.0259 × 10^3^	5.9832 × 10^3^	1.6802 × 10^3^	1.2759 × 10^3^	6.2752 × 10^2^	**1.0340 × 10** ^2^
	Rank	12	11	6	4	2	**1**
C25	Ave	2.0646 × 10^5^	2.9442 × 10^5^	1.0520 × 10^4^	4.2624 × 10^4^	8.6799 × 10^3^	**6.0679 × 10** ^3^
	Std	6.2660 × 10^4^	9.8789 × 10^4^	2.7744 × 10^3^	6.6328 × 10^3^	2.3094 × 10^3^	**6.1890 × 10** ^1^
	Rank	11	12	5	8	3	**1**
C26	Ave	6.7638 × 10^3^	1.1428 × 10^4^	4.9680 × 10^3^	6.0710 × 10^3^	3.9788 × 10^3^	**3.4510 × 103**
	Std	5.5201 × 10^2^	1.2443 × 10^3^	3.2669 × 10^2^	3.3254 × 10^2^	2.1108 × 10^2^	**6.4330 × 10** ^1^
	Rank	9	12	6	8	2	**1**
C27	Ave	3.2399 × 10^4^	2.9115 × 10^4^	9.1847 × 10^3^	8.4986 × 10^3^	4.5246 × 10^3^	**3.2770 × 10** ^3^
	Std	2.8213 × 10^3^	2.5432 × 10^3^	1.0138 × 10^3^	8.4867 × 10^2^	2.6381 × 10^2^	**4.9362 × 10** ^1^
	Rank	12	11	7	5	2	**1**
C28	Ave	1.5165 × 10^16^	1.1634 × 10^16^	5.0930 × 10^12^	2.3691 × 10^13^	1.2282 × 10^9^	**3.0941 × 10** ^6^
	Std	1.0569 × 10^16^	1.0278 × 10^16^	7.2343 × 10^12^	8.2516 × 10^13^	1.4546 × 10^9^	**3.4823 × 10** ^6^
	Rank	12	11	4	5	2	**1**
C29	Ave	5.1701 × 10^15^	6.1968 × 10^15^	2.4814 × 10^12^	2.0838 × 10^13^	3.3664 × 10^9^	**6.8307 × 10** ^6^
	Std	3.6081 × 10^15^	5.4952 × 10^15^	2.9205 × 10^12^	2.7394 × 10^13^	2.0573 × 10^9^	**4.7809 × 10** ^6^
	Rank	11	12	3	5	2	**1**

Bold is the best result of all algorithms.

**Table 15:** Memory usage of all algorithms.

Algorithms	C1	C4	C15	C27	Average Memory Occupation	Rank
DE	2.992MB	3.277 MB	3.812 MB	3.176 MB	3.314 MB	7
SSA	2.949 MB	2.906 MB	3.738 MB	3.484 MB	3.269 MB	4
SCA	3.242 MB	3.145 MB	3.559 MB	3.614 MB	3.390 MB	11
MFO	2.918 MB	3.211 MB	3.469 MB	3.079 MB	3.169 MB	1
WOA	2.992 MB	2.898 MB	3.758 MB	3.699 MB	3.337 MB	10
DBO	3.020 MB	3.156 MB	3.492 MB	3.367 MB	3.259 MB	2
POA	2.891 MB	2.895 MB	3.725 MB	3.559 MB	3.267 MB	3
SOA	2.848 MB	3.125 MB	3.516 MB	3.738 MB	3.307 MB	6
BWO	3.270 MB	2.965 MB	3.492 MB	3.594 MB	3.330 MB	9
GJO	3.098 MB	3.227 MB	3.539 MB	3.852 MB	3.429 MB	12
NGO	2.984 MB	2.891 MB	3.266 MB	3.988 MB	3.282 MB	5
MSINGO	2.988 MB	3.172 MB	3.441 MB	3.707 MB	3.327 MB	8

**Table 16:** Optimization results for T/CSD.

Algorithm	Optimal Values of Variable			Optimal Value	Rank
	d	D	*p*		
**MSINGO**	**0.05166107**	**0.35617546**	**11.36666667**	**1.2673687 × 10^−2^**	**1**
NGO	0.05187427	0.36129583	11.06666667	1.2674502 × 10^−2^	2
DE	0.05181379	0.35957038	11.16666667	1.2684364 × 10^−2^	3
SSA	0.05060498	0.32639958	13.86666667	1.3146121 × 10^−2^	7
SCA	0.05077799	0.33174986	14.03333333	1.3273039 × 10^−2^	10
MFO	0.05057397	0.32630009	13.86666667	1.3048161 × 10^−2^	6
WOA	0.05220499	0.36931603	11.36666667	1.2988707 × 10^−2^	5
DBO	0.05541281	0.4763829	10.3	1.4084970 × 10^−2^	11
POA	0.05203765	0.05203765	11.03333333	1.2751105 × 10^−2^	4
SOA	0.05826311	0.53740755	6.03333333	1.4125533 × 10^−2^	12
BWO	0.05109965	0.34071944	13.56666667	1.3256904 × 10^−2^	9
GJO	0.0542935	0.42723403	9.3	1.3172539 × 10^−2^	8

**Table 17:** Optimization results for CBD.

Algorithm	Optimal Values for Variable					Optimal Value	Rank
	$x_{1}$	$x_{2}$	$x_{3}$	$x_{4}$	$x_{5}$		
**MSINGO**	**6.01657085**	**5.3096501**	**4.49375284**	**3.50112914**	**2.15260674**	**1.3399595 × 10^0^**	**1**
NGO	6.01503616	5.3111729	4.49155737	3.50361648	2.15242352	1.3399655 × 10^0^	2
DE	6.01600697	5.30935665	4.49684451	3.49909257	2.15264729	1.3399744 × 10^0^	3
SSA	5.98316931	5.34544619	4.486311	3.52788932	2.1950092	1.3439603 × 10^0^	7
SCA	6.32362784	5.46814471	4.75418554	3.78577415	2.19996678	1.4059780 × 10^0^	10
MFO	6.06815902	5.20953467	4.68780796	3.52243504	2.20412934	1.3535849 × 10^0^	8
WOA	6.00676534	5.33296728	4.50572794	3.52452514	2.16214472	1.3436049 × 10^0^	6
DBO	5.99704925	5.31424301	4.47630367	3.51815414	2.18458645	1.3409970 × 10^0^	5
POA	7.91371491	8.03542057	6.69820596	5.82551409	4.01106876	2.0269969 × 10^0^	11
SOA	9.95270722	8.45674586	8.00232444	7.41729296	7.59498781	2.5848612 × 10^0^	12
BWO	6.18643737	5.33534609	4.5406274	3.50471167	2.19537516	1.3579799 × 10^0^	9
GJO	6.00710951	5.32070619	4.50851846	3.49083636	2.15950187	1.3407684 × 10^0^	4

**Table 18:** Optimization results for PVD.

Algorithm	Optimal Values for Variable				Optimal Value	Rank
	$T_{s}$	$T_{h}$	R	L		
MSINGO	0.77837747	0.38476523	40.32807677	199.89104391	5.8862290 × 10^₊3^	2
NGO	0.7985807	0.40136672	41.34128664	188.15374517	5.9514389 × 10^₊3^	3
**DE**	**0.77818553**	**0.38466527**	**40.32013235**	**199.99420876**	**5.8854618 × 10^₊3^**	**1**
SSA	1.0757963	0.55534384	54.70469246	73.84185279	6.9301306 × 10^₊3^	7
SCA	1.12202045	0.6128027	54.85201213	82.4779741	7.6979528 × 10^₊3^	10
MFO	1.00113629	0.49486844	51.87208913	100.97244603	6.4884200× 10^₊3^	4
WOA	1.01597595	0.54615418	50.65441508	108.48255737	6.9998825 × 10^₊3^	8
DBO	0.9185633	3.74627255	47.31901688	139.1836773	1.5813270 × 10^₊4^	11
POA	1.20890241	0.61547165	62.40219771	25.59088007	7.2397050 × 10^₊3^	9
SOA	3.58181582	14.21070275	57.06407472	56.44188856	1.4362201 × 10^₊5^	12
BWO	0.9268719	0.51659367	46.16063784	150.31386398	6.7770451 × 10^₊3^	6
GJO	1.03472148	0.52319187	53.3258698	85.35882201	6.6726795 × 10^₊3^	5

**Table 19:** Optimization results for WBD.

Algorithm	Optimal Values for Variable				Optimal Value	Rank
	h	l	t	b		
**MSINGO**	**0.19883231**	**3.33736532**	**9.19202432**	**0.19883231**	**1.6702177 × 10^0^**	**1**
NGO	0.19883228	3.33736578	9.19202436	0.19883231	1.6702178 × 10^0^	2
DE	0.1988319	3.33737493	9.19202659	0.19883236	1.6702192 × 10^0^	3
SSA	0.20228294	3.97160609	8.96072583	0.22009406	1.8715148 × 10^0^	7
SCA	0.18381673	4.07243877	9.33506699	0.21005482	1.8504610 × 10^0^	6
MFO	0.13257496	5.96536894	9.44693237	0.19852796	1.9021302 × 10^0^	8
WOA	0.18998614	3.56385495	9.2142258	0.20062402	1.7032059 × 10^0^	5
DBO	0.17553749	5.41072847	9.37121339	0.3164207	2.1657368 × 10^0^	11
POA	0.13799674	7.04317317	8.63542248	0.24363732	2.1635826 × 10^0^	10
SOA	0.3204752	4.45095148	5.2948073	0.72813159	3.4197885 × 10^0^	12
BWO	0.14684037	5.61671156	9.3375609	0.21199649	1.9755729 × 10^0^	9
GJO	0.19090334	3.52253955	9.19913179	0.19909769	1.6855842 × 10^0^	4

**Table 20:** Optimization results for SRD.

Algorithm	Optimal Values for Variable							Optimal Value	Rank
	b	m	*p*	$l_{1}$	$l_{2}$	$d_{1}$	$d_{2}$		
**MSINGO**	**3.5**	**0.7**	**17**	**8.171**	**8.252**	**3.9**	**5.5**	**1.3415265 × 10^₊3^**	**1**
**NGO**	**3.5**	**0.7**	**17**	**8.174**	**8.265**	**3.9**	**5.5**	**1.3415265 × 10^₊3^**	**1**
**DE**	**3.5**	**0.7**	**17**	**8.134**	**8.180**	**3.9**	**5.5**	**1.3415265 × 10^₊3^**	**1**
SSA	3.548	0.701	19.131	8.012	8.010	3.741	5.363	1.7946432 × 10^₊3^	10
SCA	3.535	0.7	17	8.173	8.286	3.849	5.497	1.3569351 × 10^₊3^	8
MFO	3.503	0.7	17	8.234	8.240	3.9	5.5	1.3428041 × 10^₊3^	5
WOA	3.505	0.7	17	8.189	8.237	3.878	5.493	1.3447225 × 10^₊3^	6
DBO	3.557	0.7	17.733	8.015	8.277	3.850	5.5	1.5282107 × 10^₊3^	9
POA	3.526	0.703	20.834	7.844	8.027	3.651	5.389	3.8108706 × 10^₊17^	11
SOA	3.567.	0.756	24.152	8.053	8.1004	3.776	5.414	9.8846695 × 10^₊18^	12
**BWO**	**3.5**	**0.7**	**17**	**8.201**	**8.259**	**3.9**	**5.5**	**1.3415265 × 10^₊3^**	**1**
GJO	3.505	0.7	17	8.151	8.183	3.871	5.495	1.3448399 × 10^₊3^	7

**Table 21:** Optimization results for T-bTD.

Algorithm	Optimal Values for Variable		Optimal Value	Rank
	$x_{1}$	$x_{2}$		
MSINGO	0.78866778	0.4082692	2.6389586 × 10^₊2^	2
NGO	0.78871489	0.40813758	2.6389602 × 10^₊2^	3
**DE**	**0.78867514**	**0.40824829**	**2.6389584 × 10^₊2^**	**1**
SSA	0.78884713	0.41517572	2.6463723 × 10^₊2^	8
SCA	0.79453751	0.40020516	2.6474966 × 10^₊2^	9
MFO	0.78737985	0.41270683	2.6397534 × 10^₊2^	6
WOA	0.82522025	0.36892016	2.7029955 × 10^₊2^	12
DBO	0.79085504	0.40224642	2.6391223 × 10^₊2^	5
POA	0.78794119	0.41042386	2.6390581 × 10^₊2^	4
SOA	0.79690303	0.41399512	2.6679773 × 10^₊2^	10
BWO	0.78871128	0.40914414	2.6399565 × 10^₊2^	7
GJO	0.83065798	0.36955195	2.7190075 × 10^₊2^	11

## Data Availability

Inquiries about data availability should be directed to the author.
